# Supplementary material for: Hippocampal sharp-wave ripples correlate with periods of naturally occurring self-generated thoughts in humans
Source: Nat Commun. 2024 May 22;15:4078. doi: 10.1038/s41467-024-48367-1 (PMC11111804; doi:10.1038/s41467-024-48367-1)
Supplement: Supplementary file 1 — Supplementary Information [file 41467_2024_48367_MOESM1_ESM.pdf]

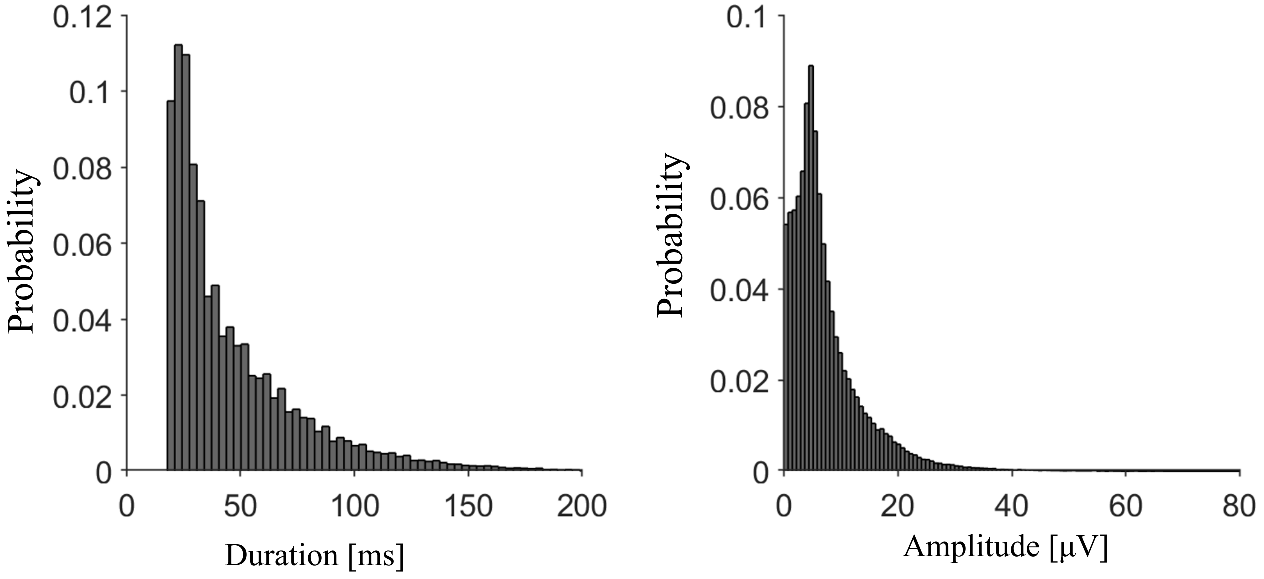

Supplementary figure 1
Distributions of identified durations (left) and amplitudes (right) of sharp-wave ripples (SWRs) in a representative patient.(n=1, Pt-04) SWR duration and amplitude both exhibited a log-normal distribution.

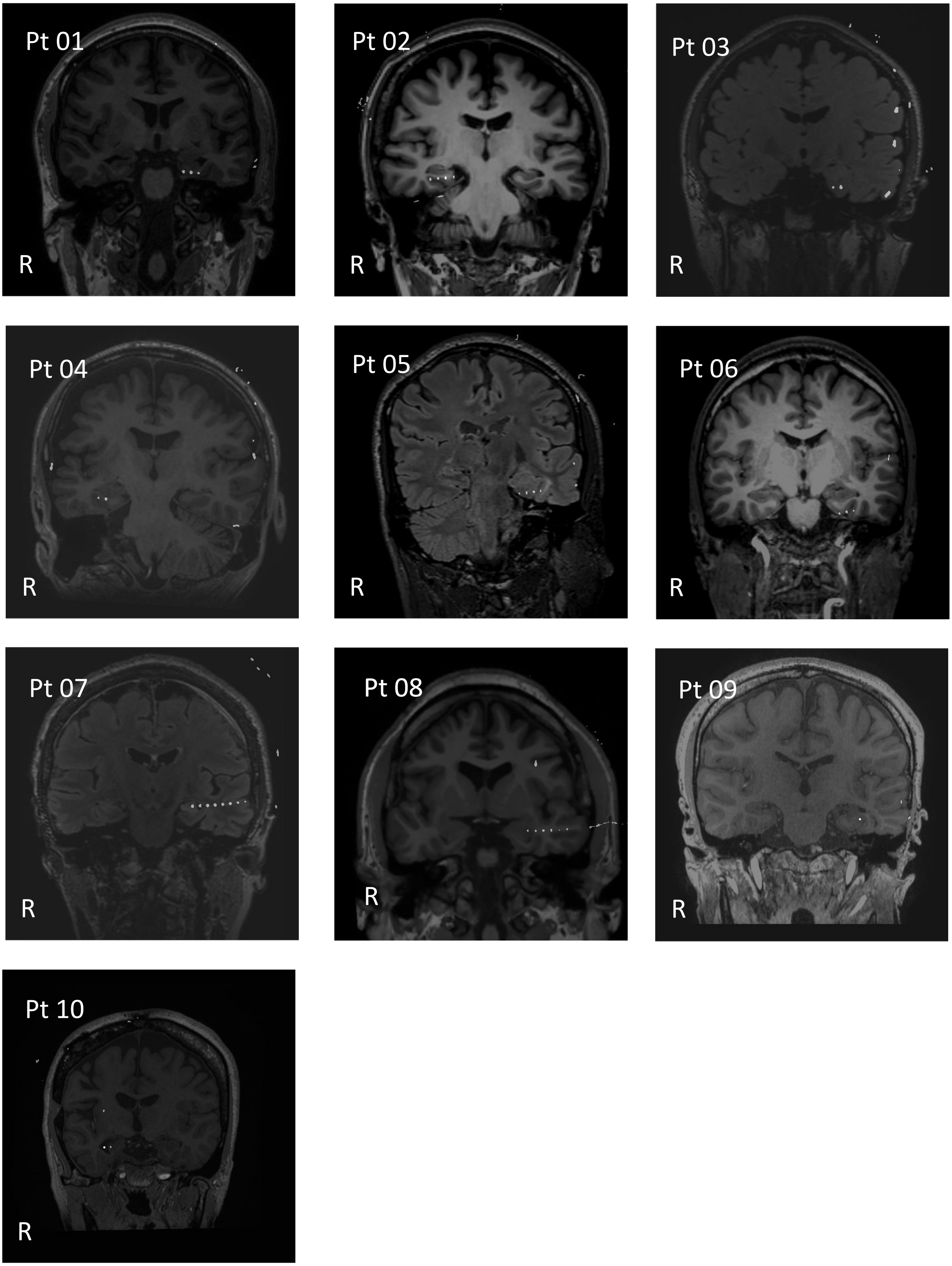

Supplementary figure 2
Combined coronal images of head using preoperative MRI data and postoperative (intracranial electrode implantation) CT data for all patients.White points indicate the position of electrodes.

a

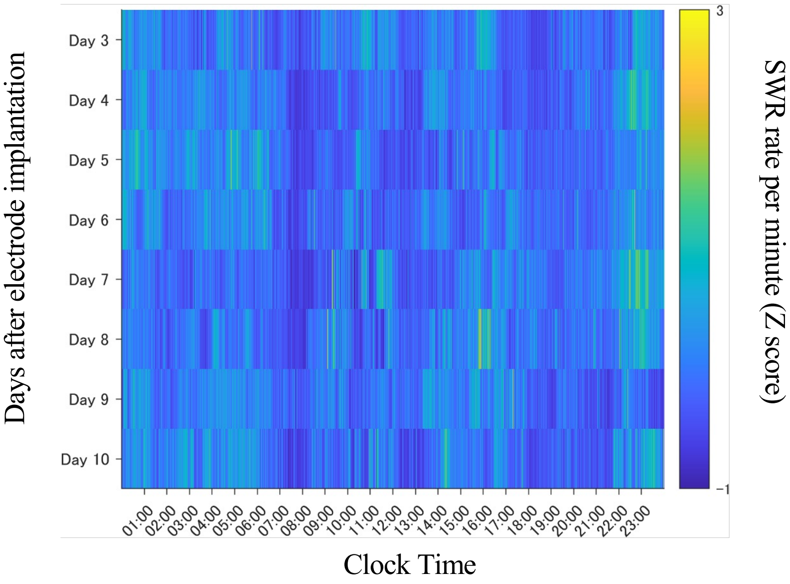

b

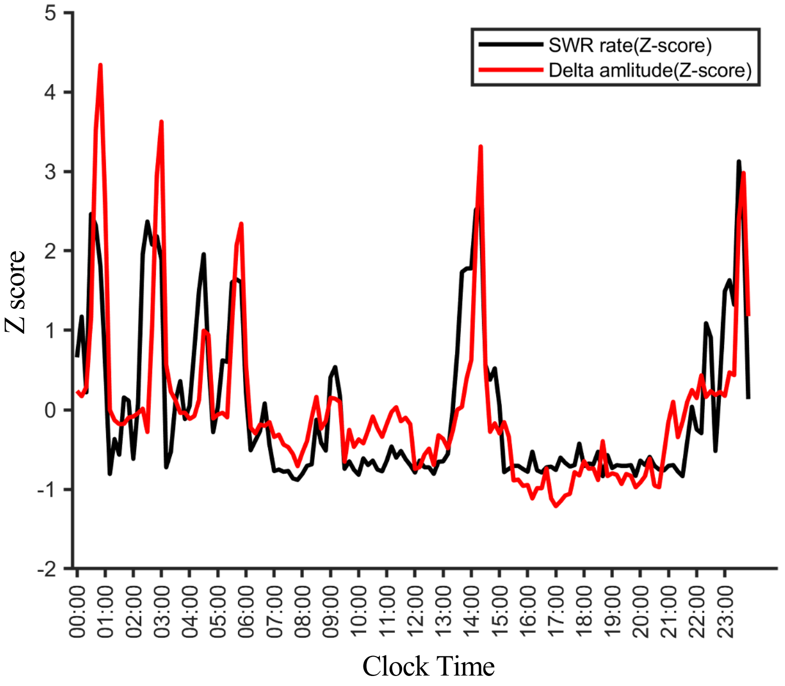

Supplementary figure 3

(a) A raster plot of the mean z-score SWR rate in all patients. The SWR rate tended to decrease during daytime and to increase during night. n=10 (b) The diurnal fluctuations of z-score SWR rates (black line) and delta amplitudes (red line), which were computed based on the recordings from cortical electrodes in a representative patient (Pt-04).

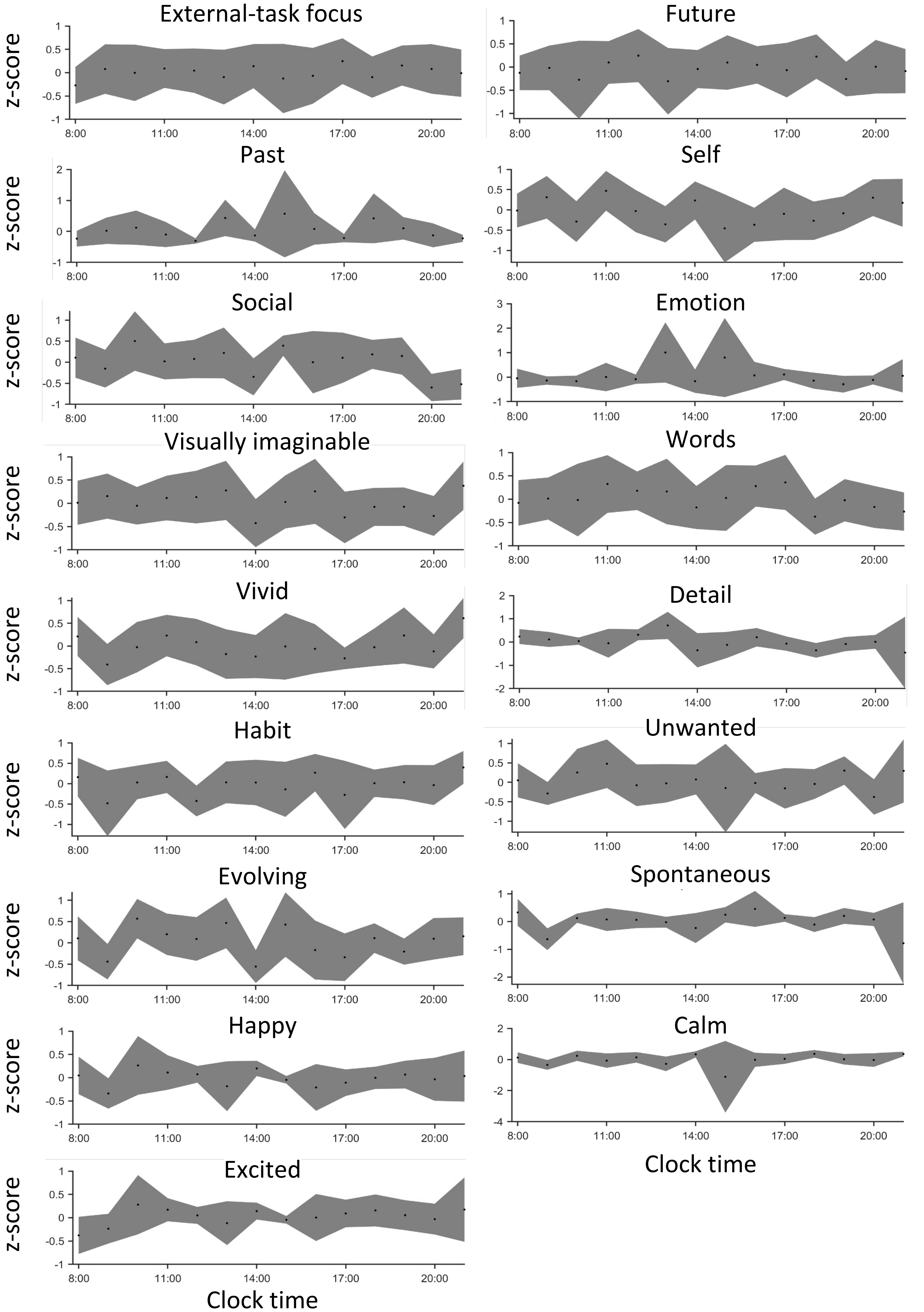

Supplementary figure 4

Diurnal fluctuation of instantaneous thoughts and moods. The black data points represent the mean, while the gray shading represents the 95% confidence interval among 10 patients. Each score for the 17 questions were z-normalized among each patient (*n*=10).

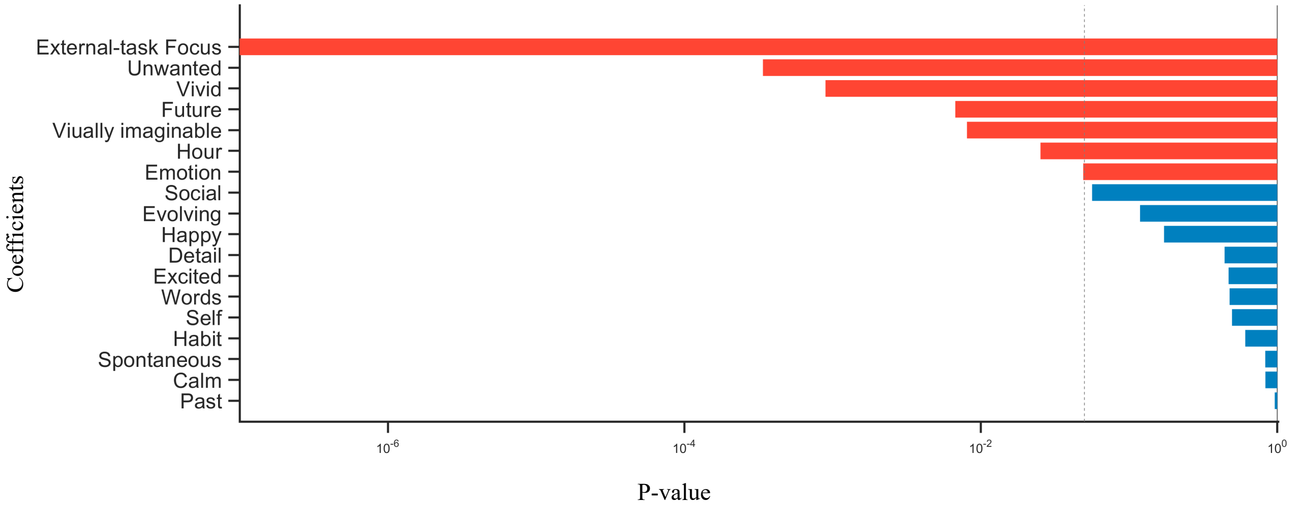

Supplementary figure 5

The *P*-value of each feature in the linear mixed-effect model used to explain the normalized SWR rates averaged between 5 minutes before to 0 minutes after the answering questions. The gray dotted line indicates *P*=0.05.

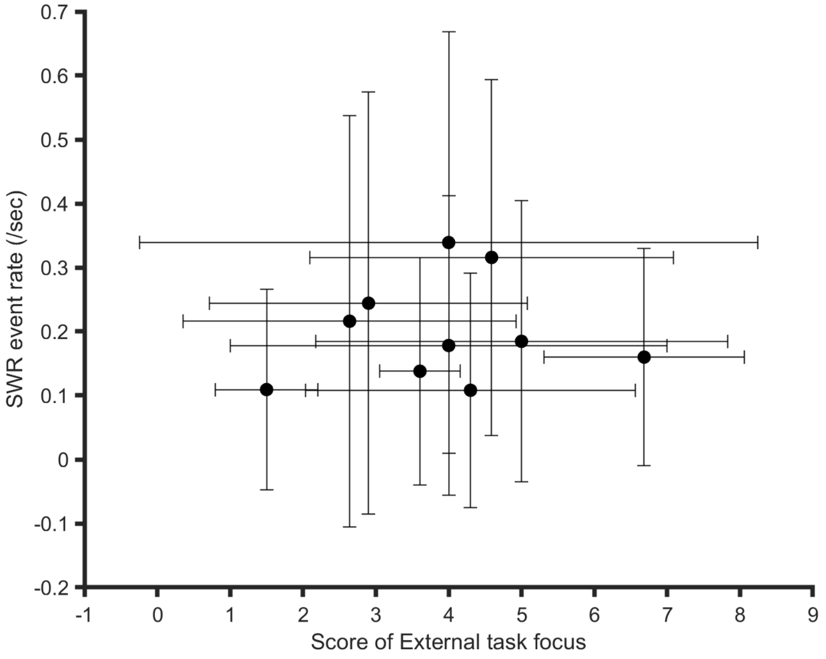

Supplementary figure 6

Plot of mean and standard deviation of SWR event rate(/s) during the day 7:00 to 21:59 and responses to the "External-task Focus" items of each patient (*n*=10). The SWR event rate was not normalized.

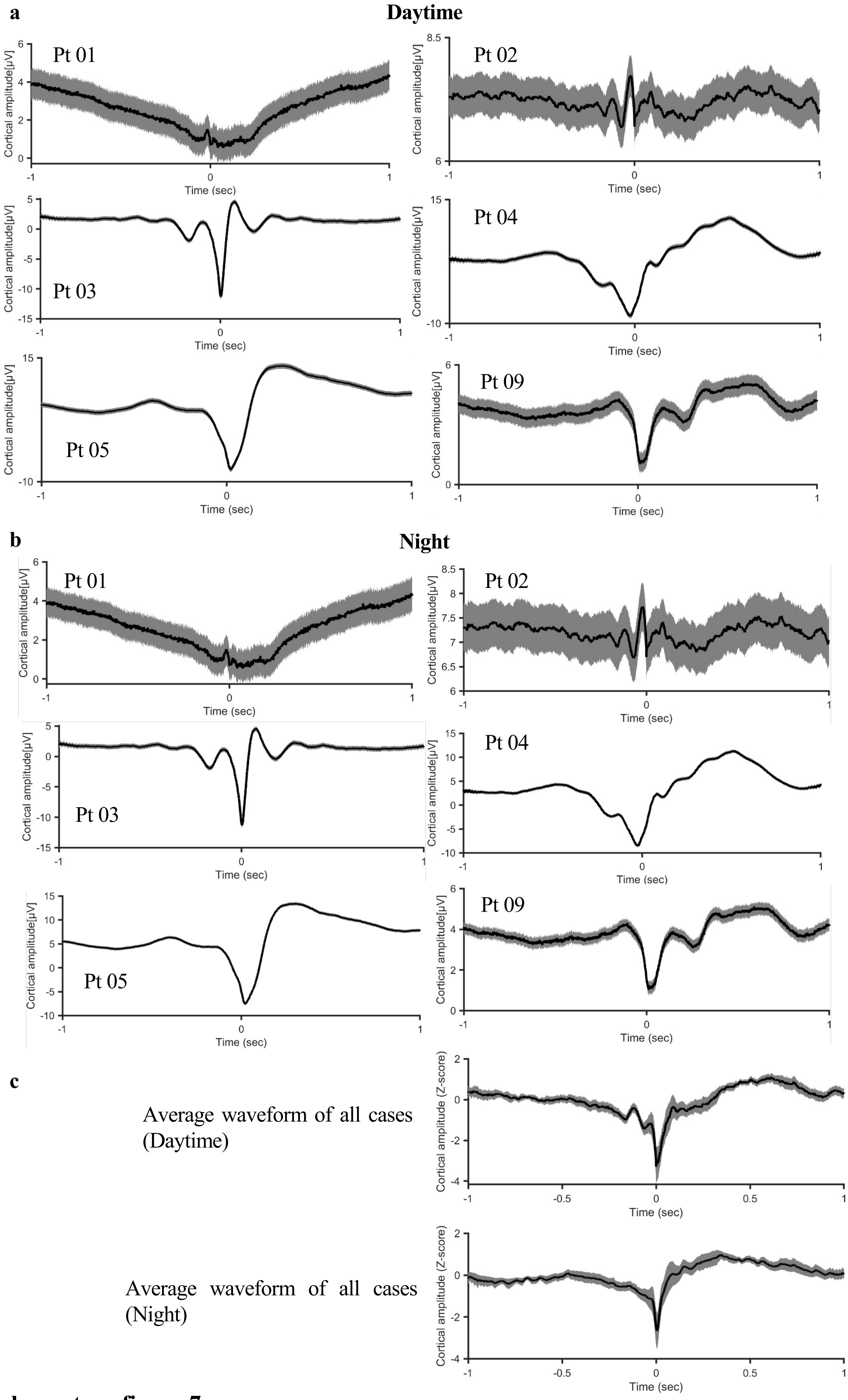

Supplementary figure 7

The average waveforms of all cortical EEG electrodes were drawn 1 second before and after the peak of SWR during both daytime (from 7:00 to 22:00, a) and night (22:00 to 7:00, b) in 6 patients who were implanted the electrodes at cortices. (c) The average waveforms among 6 patients for daytime and night. Gray shades indicate 95% confidence intervals.

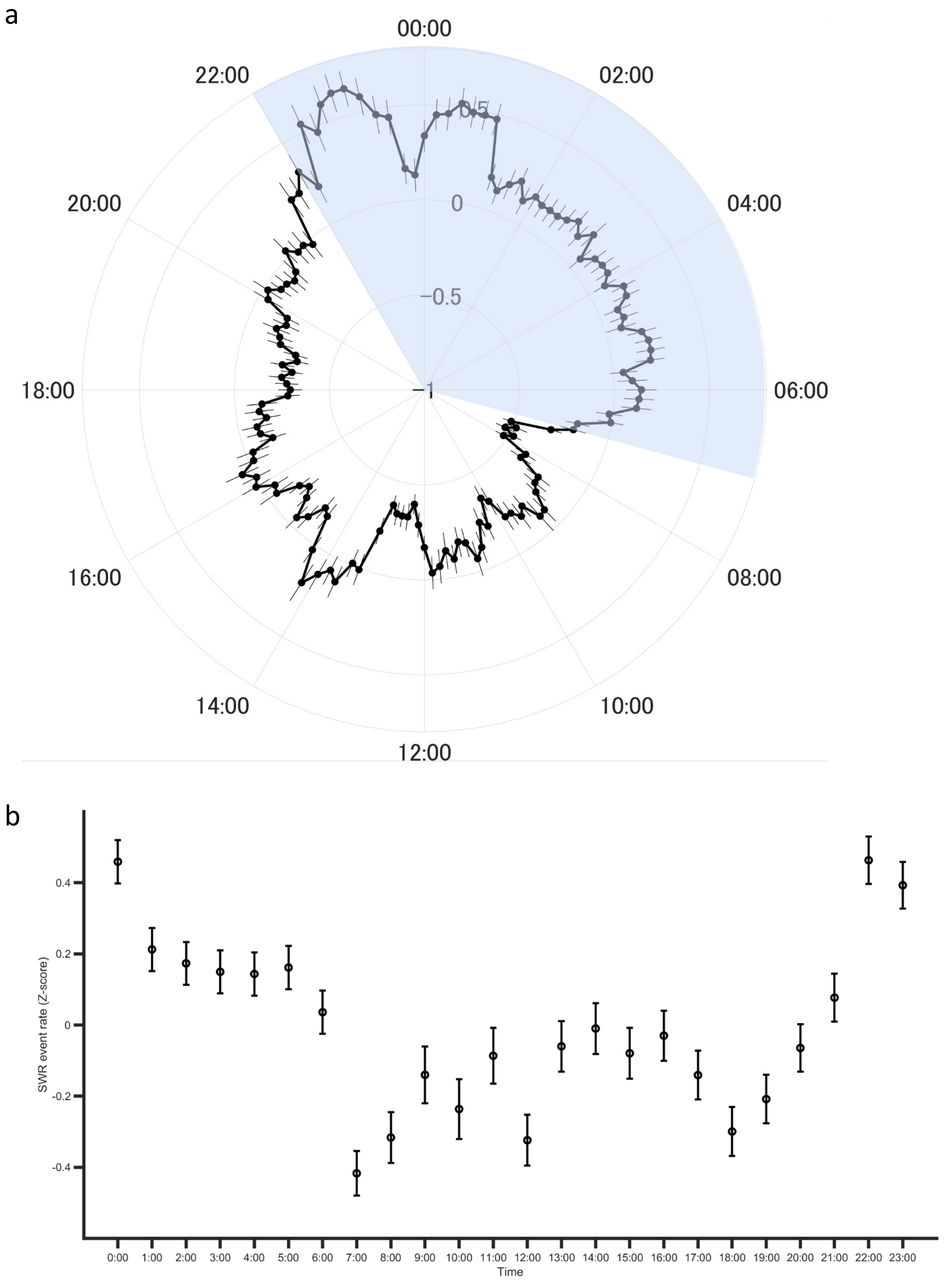

Supplementary figure 8

(a) Radial plot of diurnal fluctuation of normalized SWR rates (*n*=10). Blue shade indicates nighttime.(P<0.001, F143,83392 = 41.6, n =113760 time points from a total of 79 days in 10 patients; one-way ANOVA)(b) The post-hoc multiple comparisons of the hourly SWR event rates of all cases (*n* = 10). The circle and bar represent the mean and confidence interval for SWR event rate for one hour. The non-overlapped confidence interval among circles shows the significant difference between the circles.

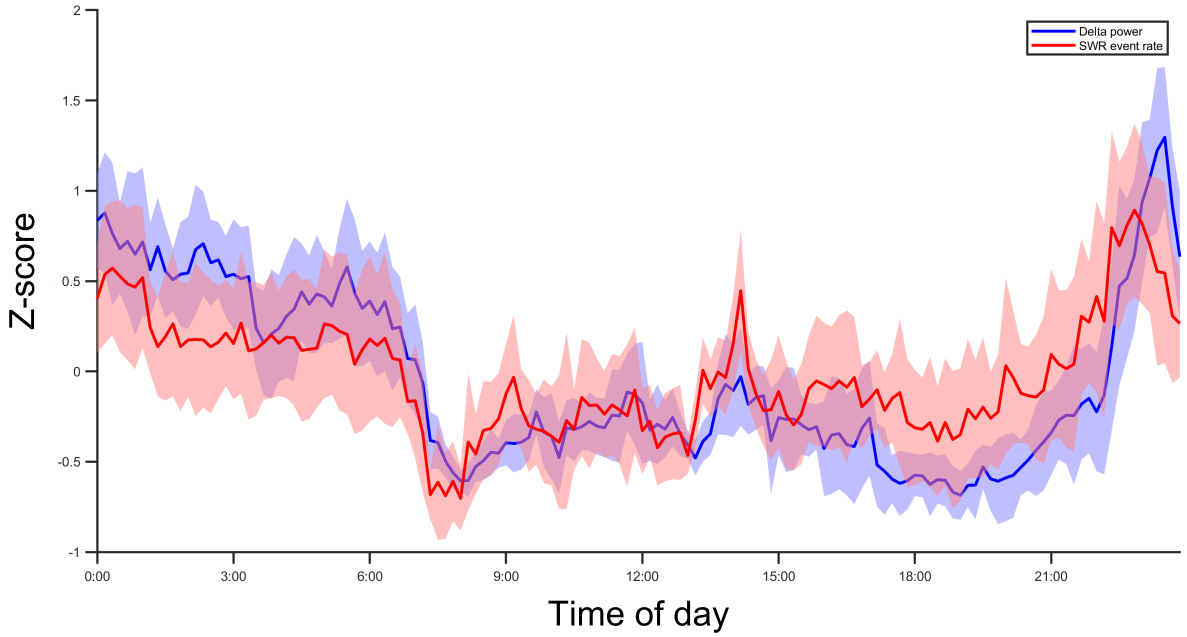

Supplementary figure 9

The mean SWR event rates and cortical delta band power of all participants in iEEG recording after day 4 are illustrated. The lines display the mean and 95% confidence interval of the Z-scored ripple event rates (red) and delta power (blue) over 24 h for all participants (n=10). The x-axis displays time and the y-axis demonstrates the Z-score of the SWR event rate or delta band power.

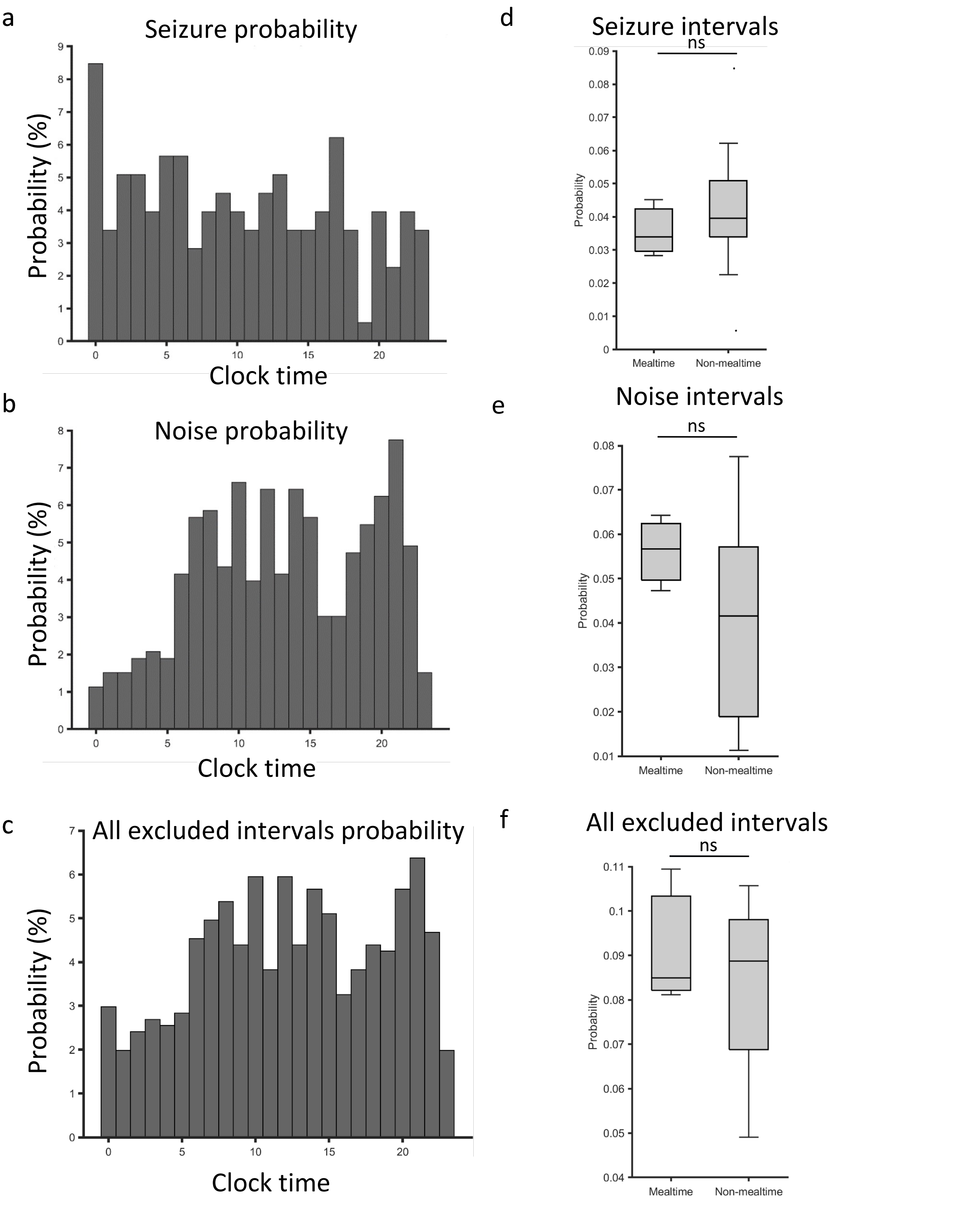

Supplementary figure 10

Histograms showing the frequency of excluded intervals at each time of day. (a) Frequency of intervals excluded as epileptic seizures. (b) Frequency of intervals excluded as artifacts. (c) Frequency of all excluded intervals. (d) The difference in the probability of excluded intervals with seizure during meals (at 7:00, 12:00 and 18:00) and non-meal times (P=0.48, n=24, Student's t-test). The frequencies were not significantly different between the mealtime and non-mealtime. (e) The difference in the probability of excluded intervals with artifacts during meals and non-meal times (P=0.19, n=24, Student's t-test). The frequencies were not significantly different between the mealtime and non-mealtime. (f) The difference in the probability of all excluded intervals during meals and non-meal times (P=0.38, n=24, Student's t-test). The frequencies were not significantly different between the mealtime and non-mealtime.

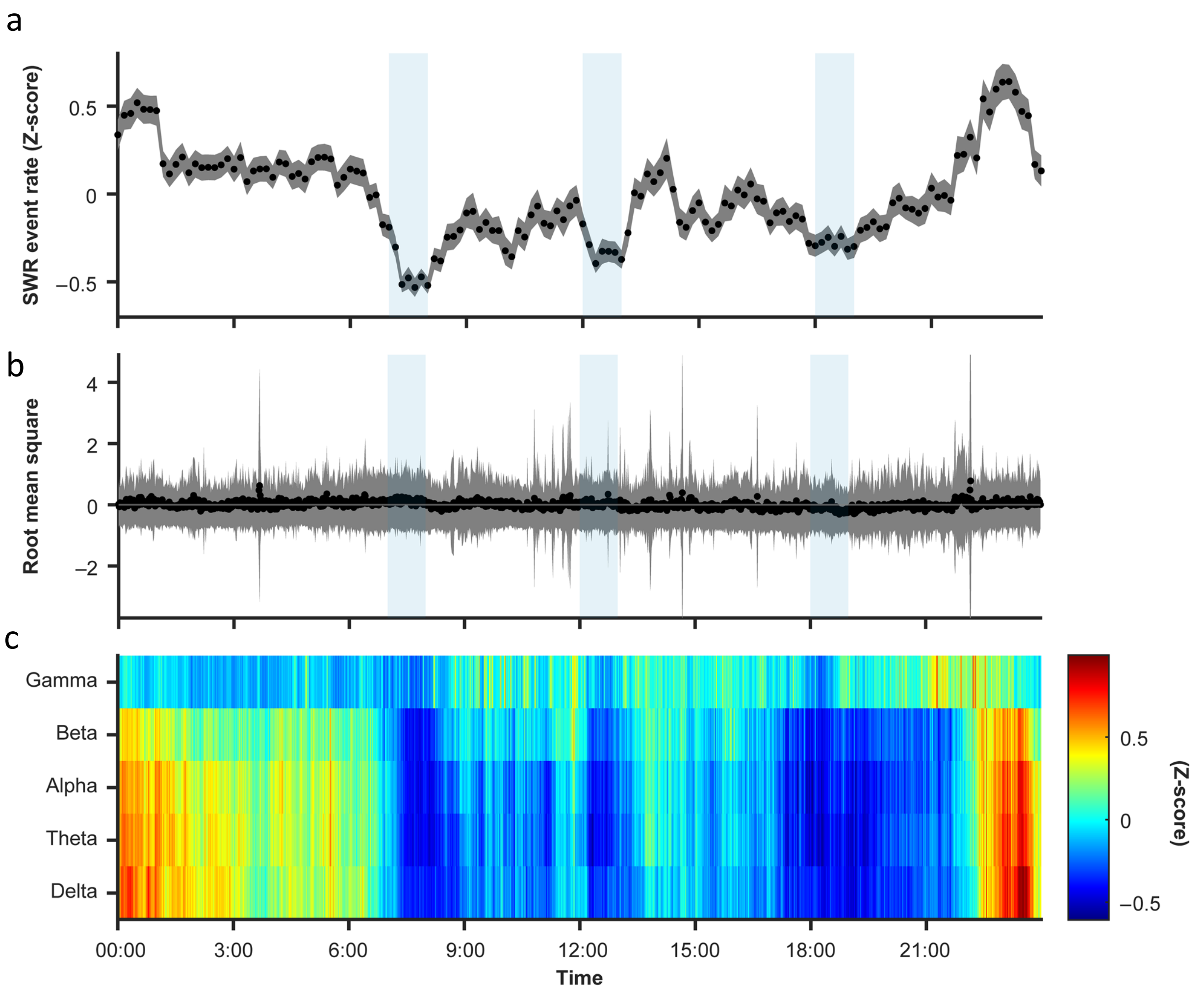

Supplementary figure 11

(a) The Z-scored SWR rate averaged for all patients at each time of day. Gray shades indicate 95% confidence intervals. Blue shades indicate mealtimes. (b) The root mean square of the signals of the hippocampal electrodes were assessed for every 1 minute and were averaged for each time of the day. The figure shows the average of the mean root mean squares among all patients. Gray shades indicate 95% confidence intervals. Blue shades indicate mealtimes. (c) The Z-normalized power spectrum for the signals of the hippocampal electrodes were averaged for each frequency band at each time for all cases (n = 10).

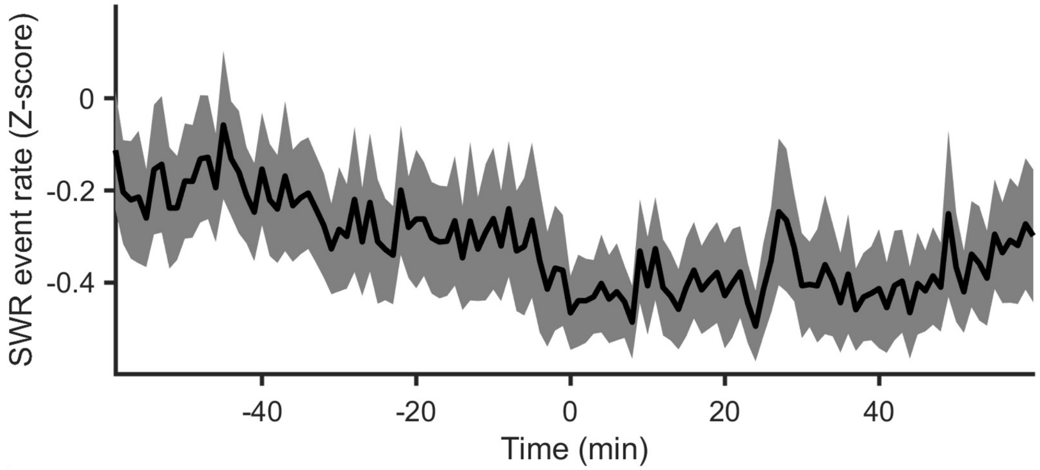

Supplementary figure 12

The mean and 95% CI (gray shade) of the Z-score of the SWR event rate for the hour before and after the start of the meal for all patients were plotted. The time to start the meal was determined by video monitoring for each patient.

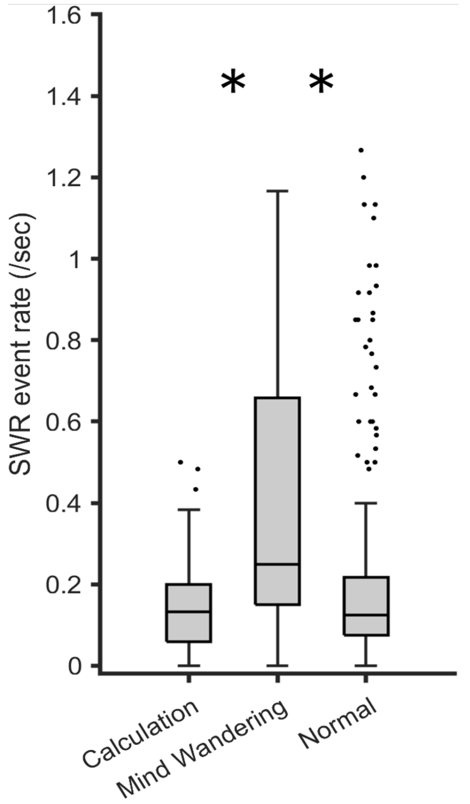

Supplementary figure 13

We did an additional experiment to assess SWR events of three new patients (5 hippocampi) when the subjects intentionally focused on breathing and when they were engaged in calculations. Focusing on breathing is a known technique to induce mind wandering. Three patients implanted electrodes in the hippocampus were instructed to focus on their breathing without controlling breathing frequency for 10 minutes. And after a rest, the same patients were instructed to do a calculation where they kept subtracting three, seven, nine, eleven or thirteen from 100 for 2 minutes each, for a total of 10 minutes. We instructed the subject to say the results of the calculation aloud each time to confirm that they are concentrating to the task. On the other hand, while the subjects were forcing on their breathing, we asked the subjects how much they focused on the task every 1 minute to assess the mind-wandering during the task. The patients rated their focus on the breathing task by the score from 1 to 5; 1 being the most focused and 5 being not focused at all. According to the subjects' reports, three patients experienced mind-wandering with the scale of 2.5 ± 1.1 during the breathing task, indicating that the breathing task induced mind-wandering states.

The figure shows box plot of the SWR event rate during the 10 minutes of calculation task (calculation), breathing task (mind-wandering) and 1-hour period before and after these task, during when the subject naturally behaved (normal). The SWR rates were significantly higher in the breathing task than calculation task and normal. These results also support our hypothesis that the SWR increases during the mind-wandering state. *, $P<0.05$, $n=360$, Wilcoxon rank sum test, Bonferroni corrected.

Supplementary discussion

This study has several limitations. The degree of mind wandering should also be assessed during the calculation task to precisely demonstrate that it is attributed to differences in mind wandering. Moreover, the SWR rates can be affected by the task itself. An important aspect to note is that the task demands are a common reason why spontaneous thought rates are reduced (for prior examples of how this manipulation of task demands is useful in elucidating mappings between cognition and brain function please see Turnbull et al., 2019, Nature Communications)

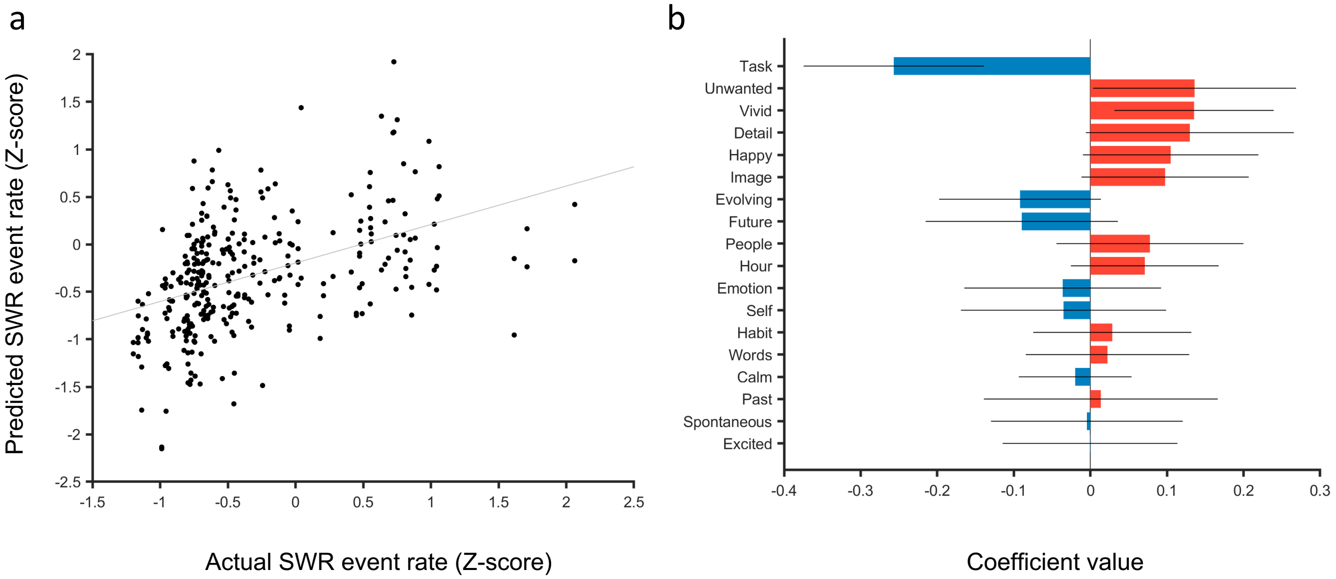

Supplementary Figure 14

A regression analysis was performed to predict the SWR event rate from the questionnaire responses in a 3 fold cross validation using a general linear regression model, keeping the order of each patient's responses. (Correlation coefficient = 0.5030, P <0.001, n = 160 answers). (a) the correlation between predicted and actual values (b) the coefficient for each cognitive characteristic. Error bars corresponding to 95% confidence intervals for the coefficients. Task, People and Image indicates "External-task Focus", "Social" and "Visually imaginable", respectively.

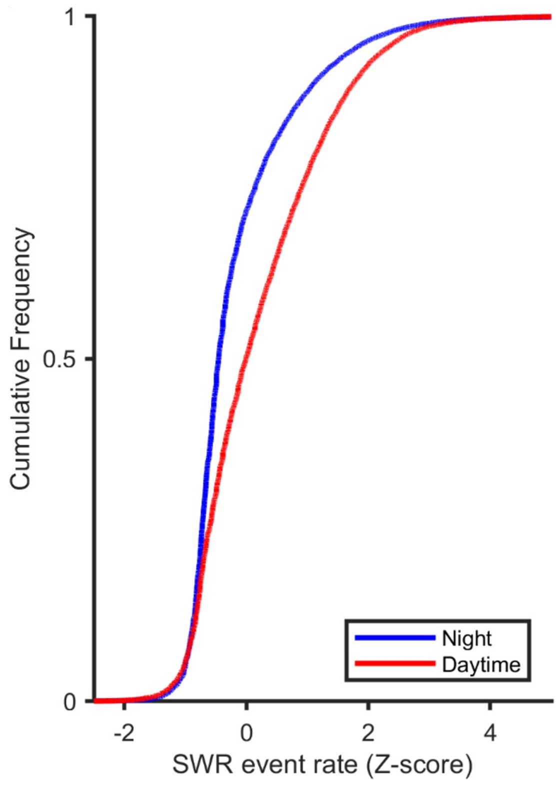

Supplementary figure 15

The cumulative frequency distribution of SWR event rates for nighttime and daytime is shown. (Blue line shows night and red line shows daytime)

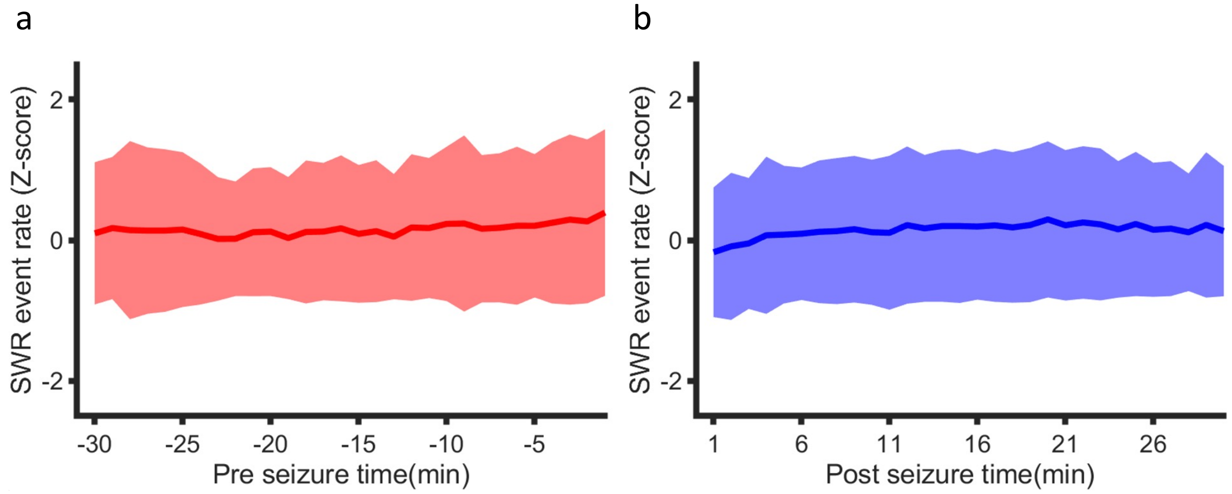

Supplementary figure 16

Plot of mean and 95% CI of SWR event rate (Z-score) for 30 minutes before and after the interval excluded as epileptic seizure (*n*=177 among all patients). The SWR rates for pre-epileptic seizure (a) and post-epileptic seizure (b) were shown with red and blue lines, respectively.

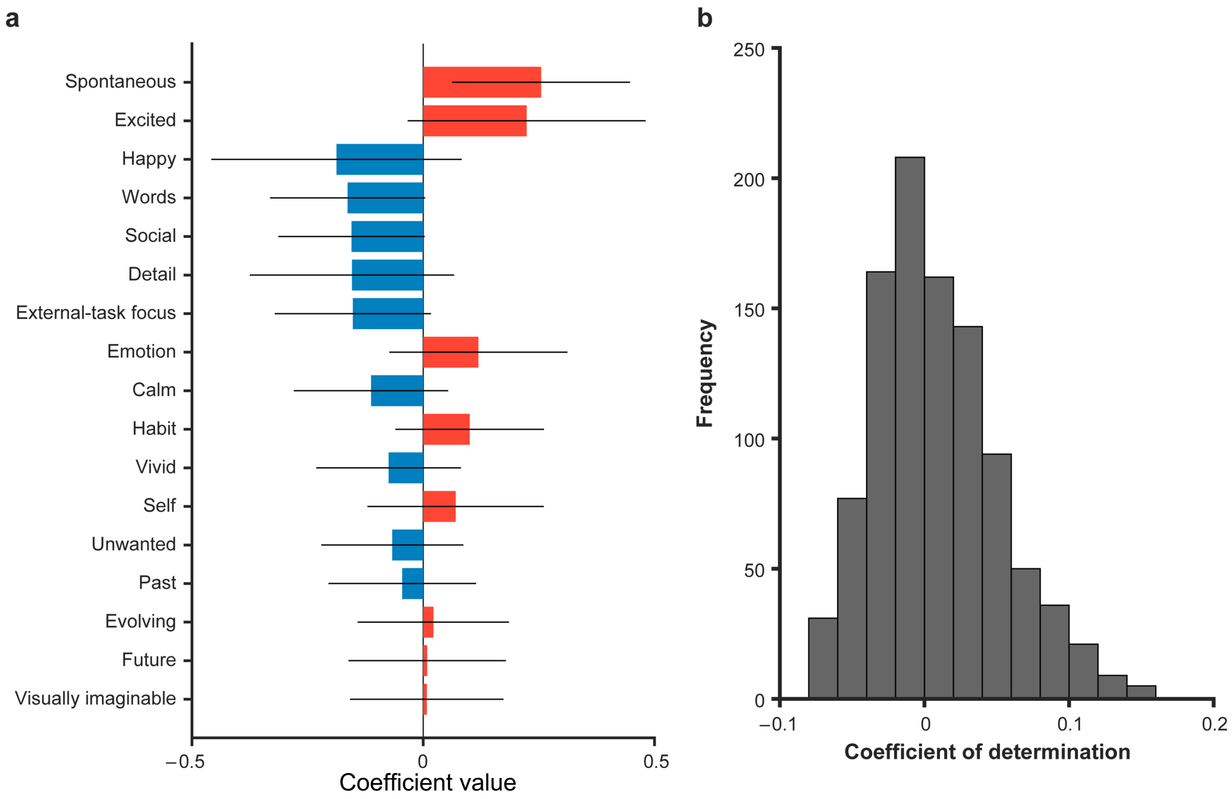

Supplementary figure 17

(a) The frequency of standardized interictal epileptic discharges (IED) of 2-7 minutes prior to questionnaire response was regressed using a mixed-effects model on the questionnaire response results (R^2=0.04). The coefficients for each cognitive characteristic are shown. Error bars corresponding to 95% confidence intervals for the coefficients. Only the item “spontaneous” contributed significantly. (b) The results of the permutation test. The order of the predicted IED frequencies was switched back and forth in random positions and regressed 1000 times to see the distribution of the coefficient of determination. The results show that the regression analysis of IED frequency is not significant ($P > 0.05$, permutation test.).

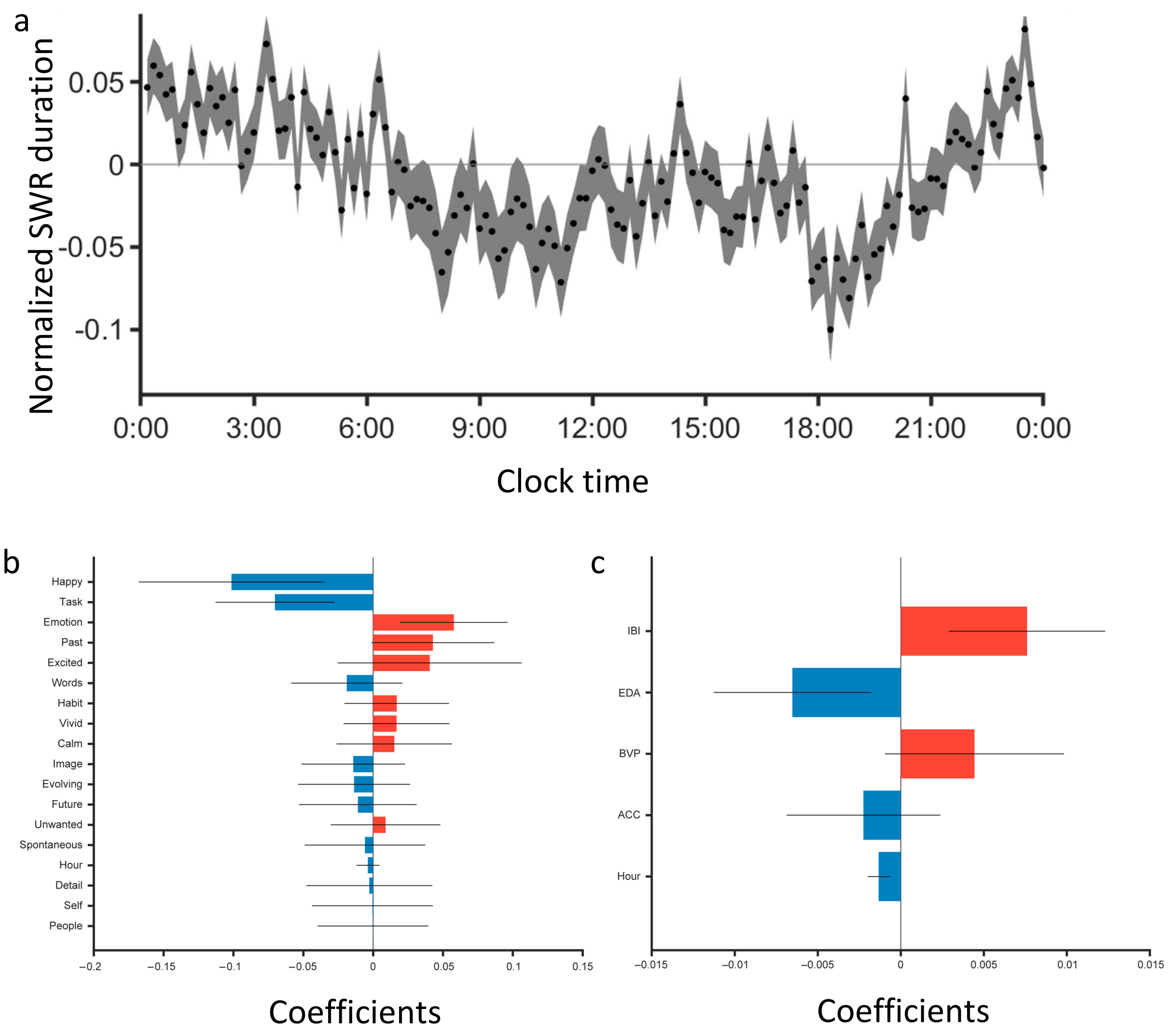

Supplementary Figure 18

(a) The diurnal fluctuation of SWR duration is shown (black points indicate mean, gray color indicates 95% CI). (b) Coefficients for each measure of regression analysis predicting standardized duration of SWR from biological data using a mixed effects model. (R^2: 0.191) Task ,People and Image indicates “External-task Focus”, “Social” and “Visually imaginable”, respectively. Error bars corresponding to 95% confidence intervals for the coefficients. (c) Coefficients for each measure of regression analysis predicting standardized duration of SWR from biological data using a mixed effects model. (R^2: 0.0066) Error bars corresponding to 95% confidence intervals for the coefficients.

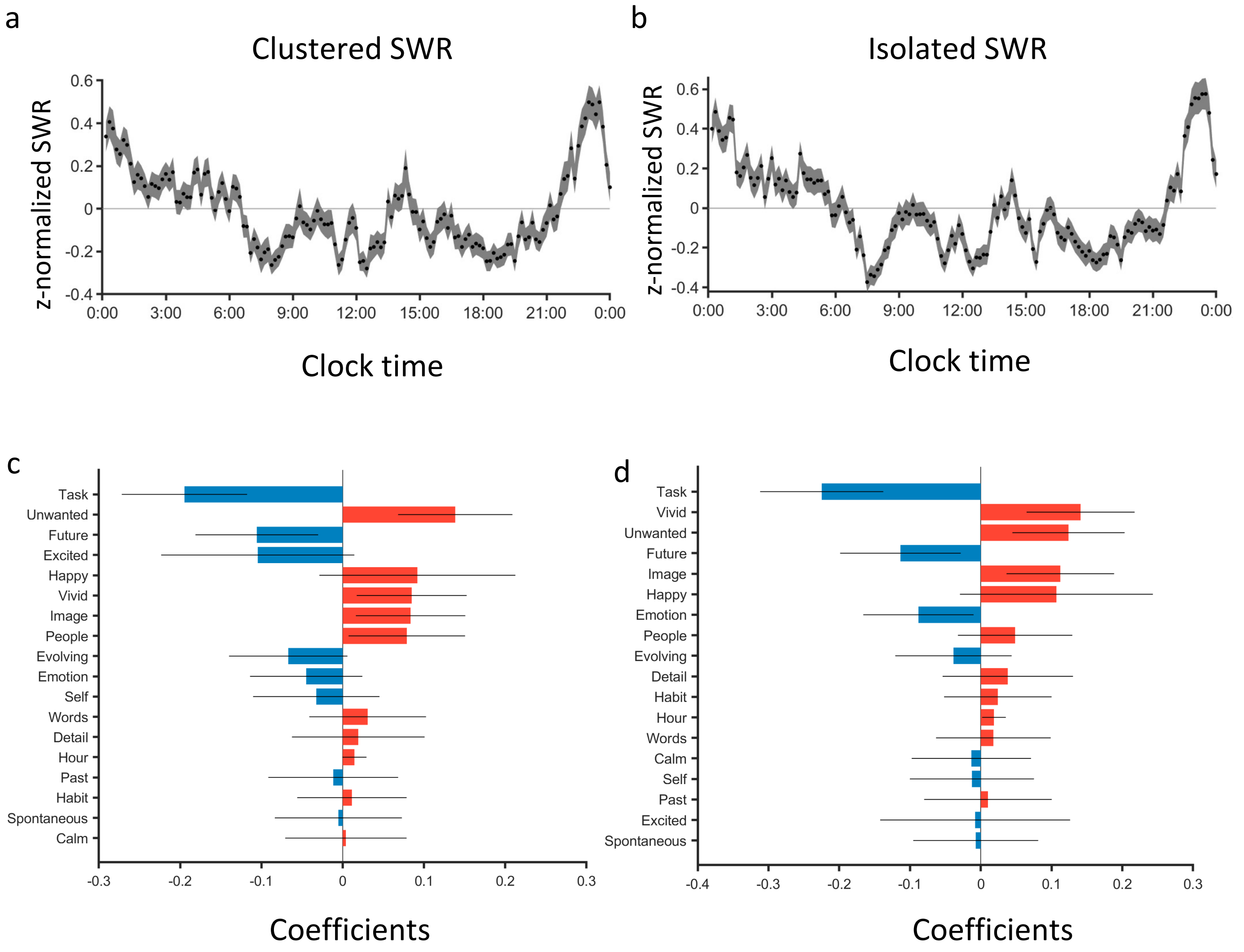

Supplementary figure 19

Diurnal fluctuations in frequency were shown when the interval between SWRs was 250 msec or less as clustered SWR and those above as isolated SWR.(a: clustered SWRs b:isolated SWRs). The normalized SWR event rates were explained by 17 scores of questionnaires using the mixed-effect model (R^2 :0.485; clustered SWR (c) and R^2 :0.567; isolated SWR (d)). Task ,People and Image indicates “External-task Focus”, “Social” and “Visually imaginable”, respectively. Error bars corresponding to 95% confidence intervals for the coefficients.

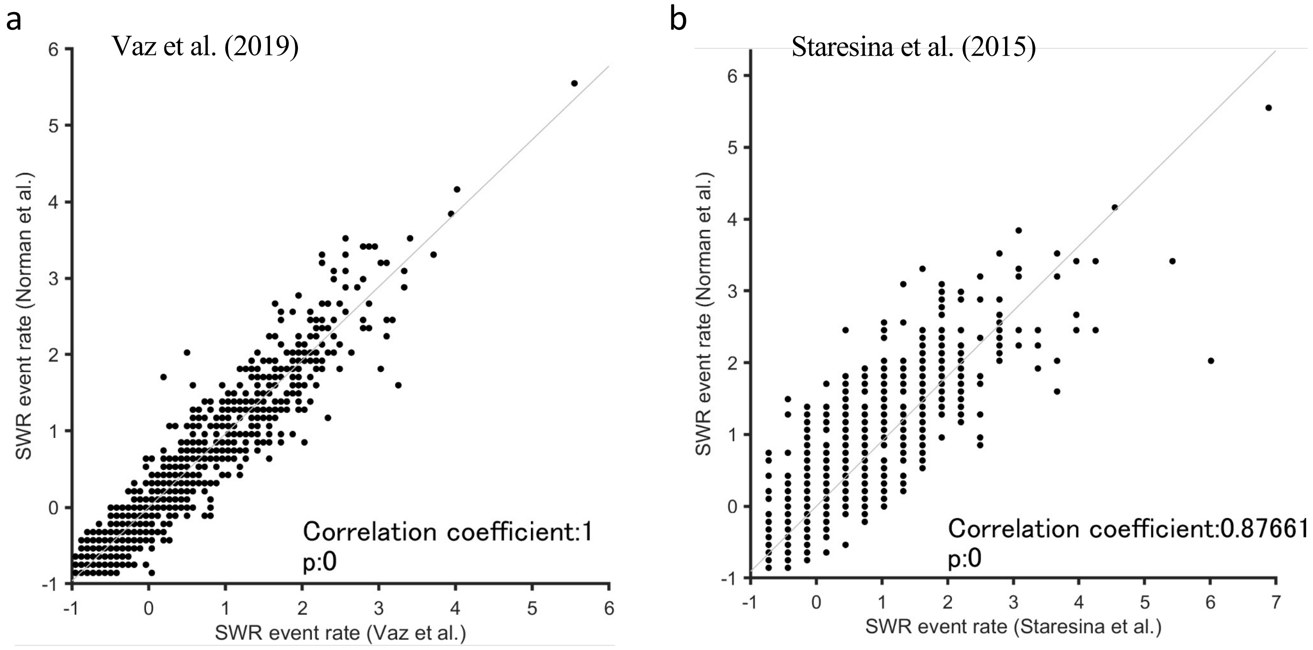

Supplementary figure 20

Comparison of the 24-hour standardized SWR rate of a representative case (Pt-03) detected by the original method (Norman et al.) with the standardized SWR rates detected by two other previously reported methods (a, Vaz et al.; b, Saresina et al.) showed a significant correlation. (Pearson's correlation)

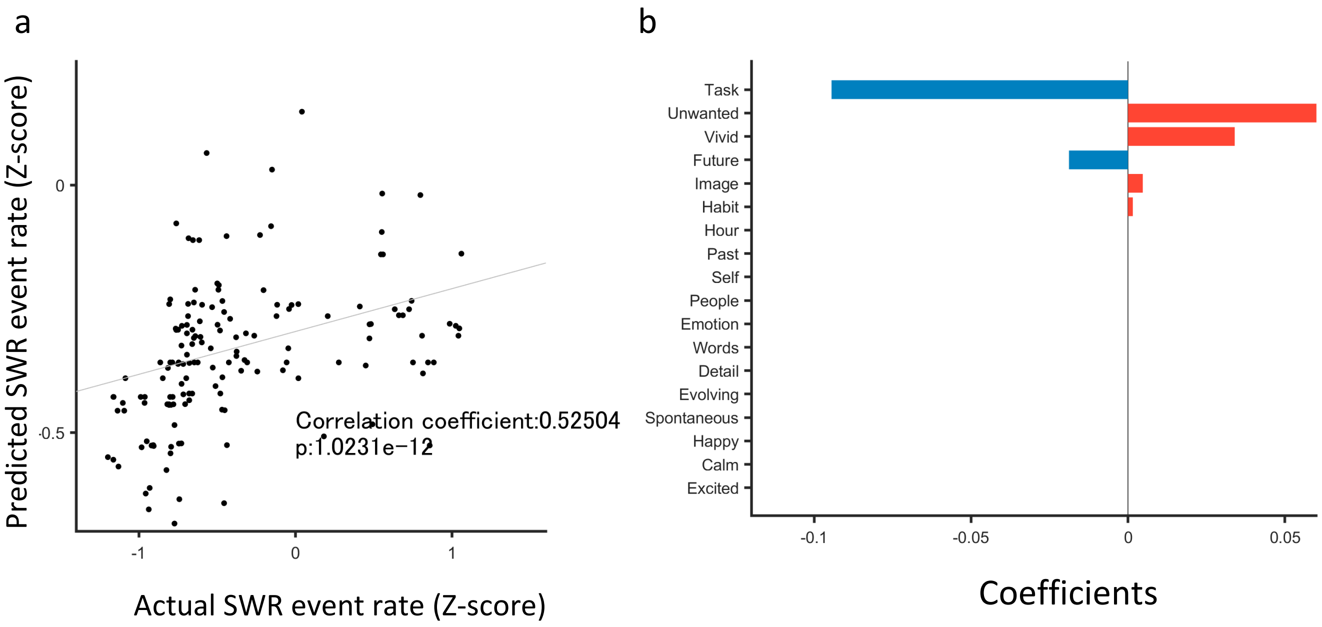

Supplementary figure 21

(a)Predictions and actual standardized SWR rate of regression analysis predicted from responses to questions about emotions and thoughts using Lasso regression. (b)The coefficient of each feature in the regression model. Task, People and Image indicates “External-task Focus”, “Social” and “Visually imaginable”, respectively.

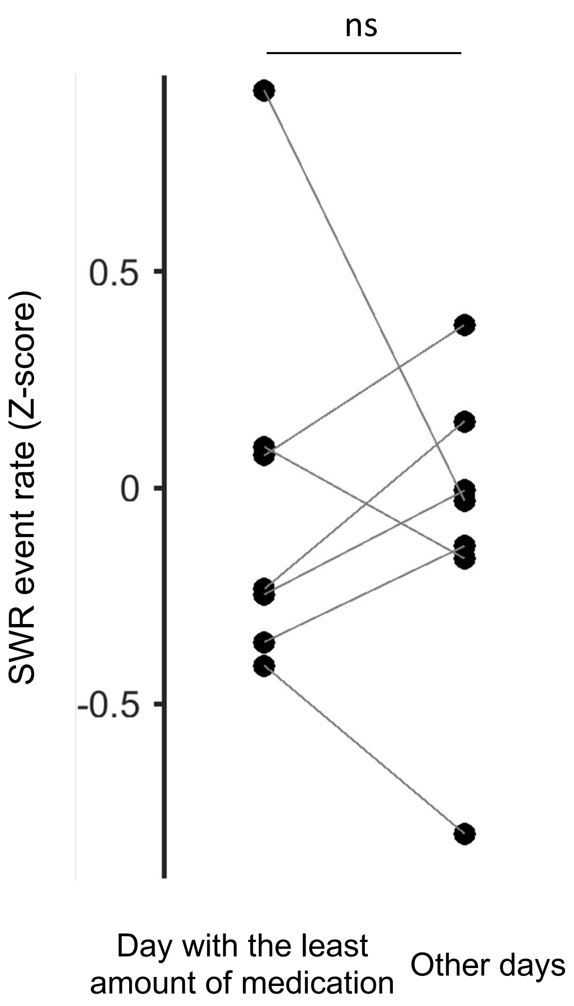

Supplementary figure 22

SWR rates on the day with the lowest dose of antiepileptic drugs were compared with those on other days, and there was no significant difference.(P = 0.74, t_6 = 0.34, n= 7 subjects; paired t-test)

Subjects with no increase or decrease in antiepileptic drugs were excluded.

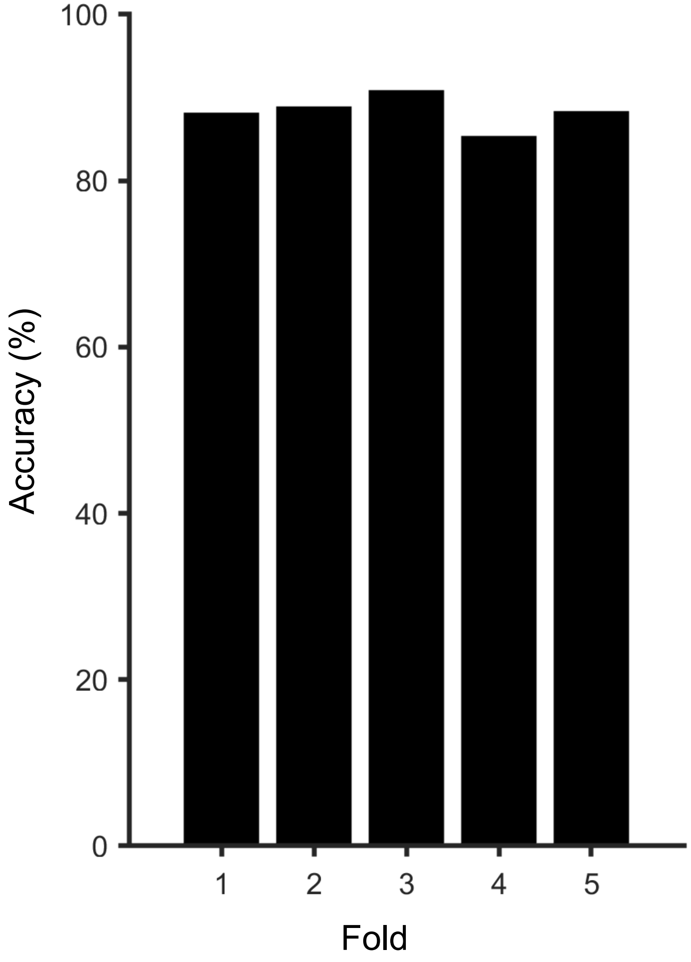

Supplementary figure 23

We classified two states in a sleep wake cycle based on four values from the wearable data (EDA, ACC, BVP, IBI): time during meals (7:00-8:00, 12:00-13:00, 18:00-19:00), which corresponds to wake period, and the period with delta power exceeding the 4 SD during the night (from 22:00 to 7:00), which corresponds to sleep period. We selected the second period as the time for deep sleep. Four values of the wearable data were averaged for each time period and normalized among all data. Two time periods were classified from the normalized values using the support vector machine in a 5-fold cross-validation. The mean classification accuracy was 88.38%.

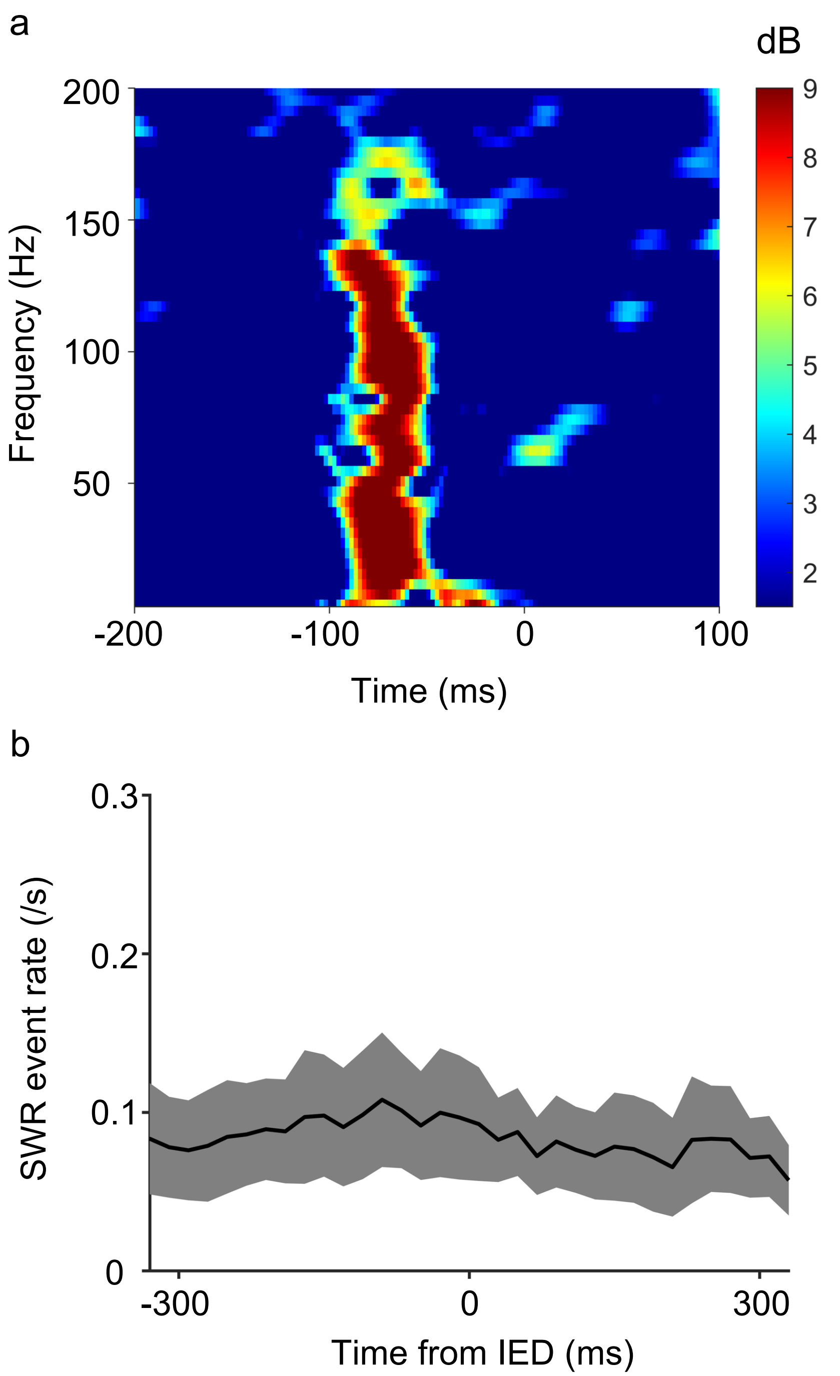

Supplementary figure 24

(a) Time-frequency map around interictal epileptic discharges (IEDs) before answering the questionnaire. Time 0 corresponds to the timing of IED onset, which was annotated manually by an epileptologist. (b) The frequency of SWR events was evaluated for iEEGs from 7 to 2 min before answering the questionnaire. The frequency was assessed in 300-ms time bins with 80% overlap from -300 to 300 ms after the onset of IED. The mean and 95% CI values are presented as black lines and shaded areas, respectively.

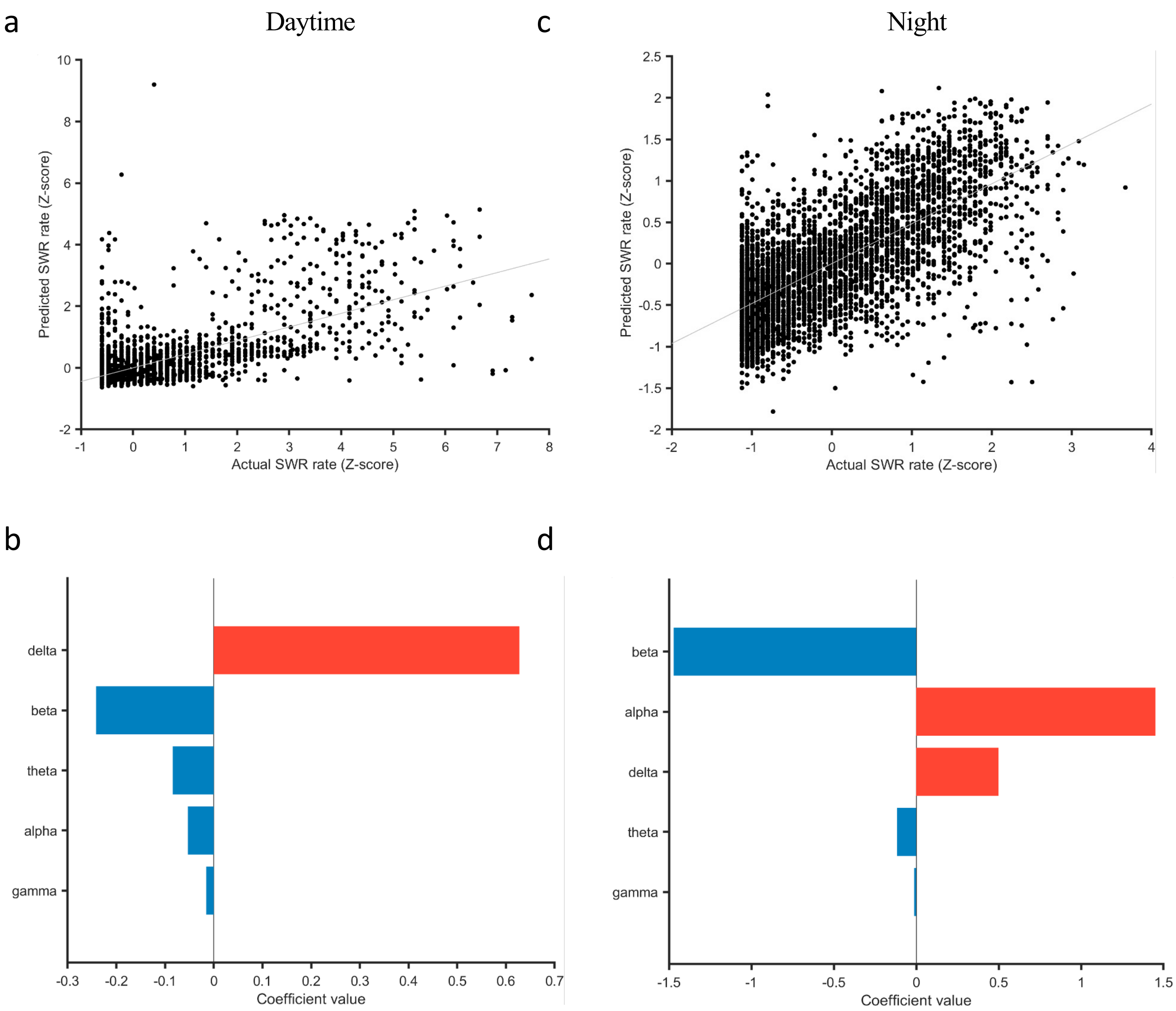

Supplementary figure 25

The normalized SWR event rates were explained by five frequency band amplitudes (theta, 4-8 Hz; alpha, 8-13 Hz; beta, 13-25 Hz; gamma, 80-150 Hz) using linear regression model for a representative case (Pt-04). (a) The actual and predicted normalized SWR event rates during the daytime were plotted with the linear regression model (R^2=0.44 ± 0.01, correlation coefficients between actual and predicted normalized SWR event rates was 0.21). (b) The coefficients of the trained model for the daytime. (c) The actual and predicted normalized SWR event rates during the night were plotted with the linear regression model(R^2=0.48 ± 0.04, correlation coefficients between actual and predicted normalized SWR event rates was 0.67).

(d) The coefficients of the trained model for the night.

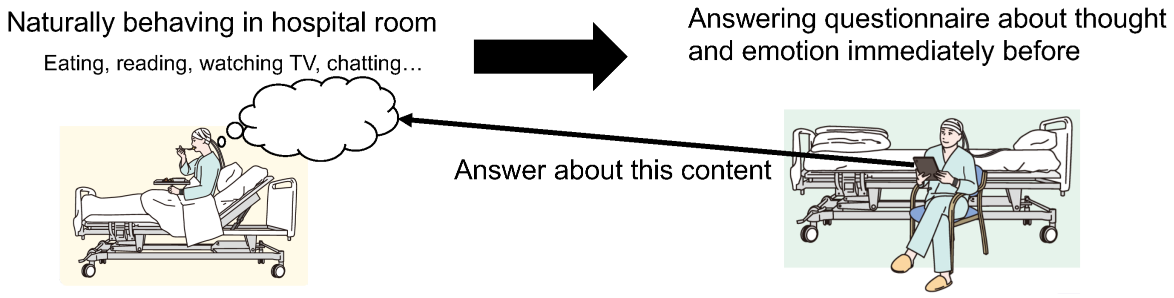

Supplementary figure 26

As is standard in mDES studies, when we refer to the 'task' in our questionnaires, we are specifically inquiring about the activity that patients were engaged in immediately before their questionnaire response. For example, if the patient was watching TV immediately before answering the questionnaire, the reported task is 'watching TV'. In addition, for example, when patients watch a TV program that is unrelated to summer vacation, their minds may inadvertently drift towards thinking about their summer vacation plans. In such instances, they should report not being focused on the task at hand or experiencing mind wandering. The tasks performed by patients immediately before answering the questionnaires varied and included diverse everyday activities, such as eating meals, reading books, and watching SNS. This figure was designed by MEDICAL EDUCATION INC. under a Creative Commons Attribution 4.0 International license.

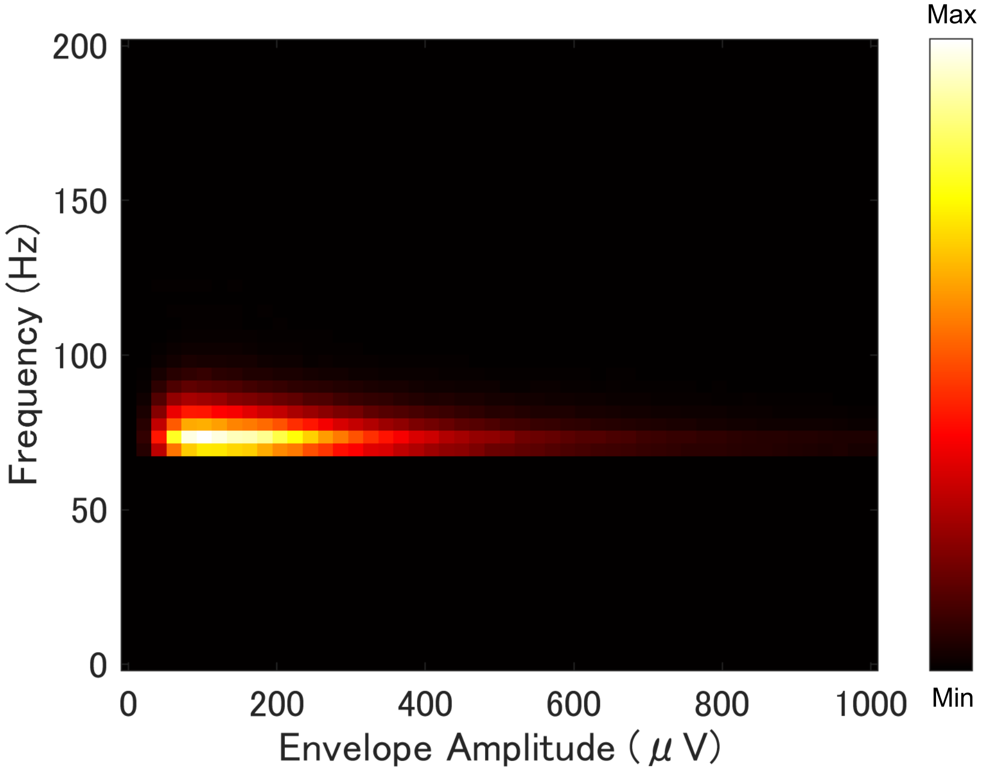

Supplementary figure 27

To assess the pathology of detected ripples, the method outlined by Ewell et al. (2019) was applied to our dataset ($N = 10$). Peak slow wave amplitude and peak frequency were determined for every ripple event. To measure the slow wave amplitude, segments of data lasting 500 milliseconds, centered on the ripple event, were band-pass filtered between 0.2 Hz and 40 Hz, and the maximum absolute amplitude of the filtered data was taken. For peak frequency calculation, the fast Fourier transform was applied to the data segment. The highest power peak for frequencies greater than 70 Hz was identified as the peak frequency. Density plot reveals that all events were included in a single cluster of similar frequency and envelope amplitude.

Supplementary Table 1. Patient characteristics and information on implanted electrodes and questions

ID	Age	Electrode position	Implant duration (Days)	Number of questionnaires answered
Pt-01	20s	Left HC	14	19
Pt-02	40s	Right HC	12	10
Pt-03	10s	Left PHC	14	25
Pt-04	40s	Right PHC	14	10
Pt-05	20s	Left PHC	11	5
Pt-06	20s	Left HC	11	2
Pt-07	50s	Left HC	15	1
Pt-08	50s	Left HC	9	33
Pt-09	10s	Left HC	9	78
Pt-10	30s	Right HC	12	3

HC: hippocampus, PHC: parahippocampal cortex

Supplementary Table 2. Imaging findings, seizure focus, and semiology of epileptic seizures of each patient

Patient ID	MRI	PET	Scalp EEG	Seizure focus
Pt-1	Bilateral hippocampal atrophy and high FLAIR intensity after herpes encephalitis	Decreased uptake in the bilateral medial temporal lobes - temporal pole	Rhythmic wave starting at F7 during seizure	Left tip of temporal lobe
Pt-2	Right temporal lobe cortical dysplasia	Decreased uptake in the lateral right temporal lobe	interictal spike at P4,02,T4,T6	Right lateral temporal lobe
Pt-3	Left hippocampal atrophy	Decreased uptake in the tip and medial portions of the left temporal lobe	Rhythmic wave starting at F7T3 during seizure	Left medial and lateral temporal lobe
Pt-4	Left hippocampal atrophy, right normal	Decreased apical in the left hippocampus	Interictal spikes at F8T4 and F7T3	"Left" medial temporal lobe
Pt-5	Cystic lesion in left fusiform gyrus	Decreased apical around the lesion	Interictal epileptic discharges at T3T5	left spindle gyrus
Pt-6	FLAIR high intensity in the lateral inferior horn of the left lateral ventricle	Decreased uptake in left frontal, parietal, and temporal cortices, with no difference in hippocampus between left and right	High amplitude spikes at left temporal	unknown
Pt-7	Posthemorrhagic changes in left parietal lobe, left hippocampal atrophy	Decreased uptake in the medial portion of the left temporal lobe	Rhythmic wave starting at F7T3 during seizure	Left supramarginal gyrus to posthemorrhagic change lesion in parietal lobe
Pt-8	Encephalocele s/p at the tip of left temporal lobe, suspected heterotopia in left ventricle	Decreased uptake in anterior left temporal lobe and left posterior parieto-parietal lobe border area	Interictal spike and waves at F7T3	Brain mass in the tip of left temporal lobe
Pt-9	T2 high intensity in medial temporal lobe	No data	Interictal spikes at T3	Parahippocampal gyrus, amygdala
Pt-10	After resection of right frontal lobe	Decreased uptake in right temporal lobe to lateral parietal lobe	Interictal spikes at T4 and F8,. F8-onset rhythmic wave during seizure	Unknown

Supplementary Table 3. Information on the amount of anti epileptic drug and food intake for each patient

Pt ID		Preoperation	Day 1	Day 2	Day 3	Day 4	Day 5	Day 6	Day 7	Day 8	Day 9	Day 10	Day 11	Day 12	Day 13
	LEV	1000	500	1000	1000	750	500	750	1000	→	→	→	→	→	→
	LTG	200	→	→	→	→	→	→	→	→	→	→	→	→	→
	LCM	400	0	→	→	→	→	→	→	→	→	→	→	→	→
	CLB	10	→	→	→	→	→	→	→	→	→	→	→	→	→
1	Breakfast	Main dish	1	1	4	6	4	6	6	6	6	6	6	6	6
		Side dish	2	2	6	6	6	6	6	6	6	6	6	6	6
	Lunch	Main dish	1	3	6	6	6	6	6	NaN	6	6	6	6	NaN
		Side dish	1	3	6	4	6	6	6	NaN	6	6	6	6	NaN
	Dinner	Main dish	1	3	6	4	6	6	6	6	6	6	6	6	6
		Side dish	1	6	6	4	6	6	6	6	6	6	6	6	6
	LEV	1000	→	→	→	→	→	→	→	→	→	→	→		
	CBZ	450	→	→	→	→	→	→	→	→	→	→	→		
	VPA	700	→	→	→	→	→	→	→	→	→	→	→		
	ZNS	300	→	→	→	→	→	→	→	→	→	→	→		
	CLB	30	→	→	→	→	→	→	→	→	→	→	→		
2	Breakfast	Main dish	1	1	5	4	1	6	4	6	6	6	6		
		Side dish	6	6	6	6	5	6	5	6	6	6	6		
	Lunch	Main dish	4	4	2	6	2	6	1	6	6	6	6		
		Side dish	2	4	4	6	5	6	6	6	6	6	4		
	Dinner	Main dish	4	4	1	1	6	2	1	6	6	1	6		
		Side dish	4	4	6	4	6	5	6	6	6	6	6		
	LEV	2500	2000	→	→	→	1500	1000	→	→	→	2500	→	→	→
	LCM	250	→	200	→	250	150	→	→	→	→	250	→	→	→
	CLB	3	→	→	→	→	→	→	→	→	→	→	→	→	→
	Breakfast	Main dish	6	6	6	6	6	6	6	6	6	6	6	6	6
3		Side dish	6	6	6	6	6	6	6	6	6	6	6	6	6
	Lunch	Main dish	4	6	6	6	6	6	6	6	6	6	6	6	6
		Side dish	6	6	6	6	6	6	6	6	6	6	6	6	6
	Dinner	Main dish	6	6	6	6	6	6	6	6	6	6	6	6	6
		Side dish	6	6	6	6	6	6	6	6	6	6	6	6	6

LEV: levetiracetam**,** LTG: lamotrigine, LCM: lacosamide, CLB: clobazam, CBZ: carbamazepine, VPA: valproic acid, ZNS: zonisamide, PER: perampanel**.** The numbers indicated in the antiepileptic drug column represent the daily dosage in milligrams (mg). The numbers indicated in the meal intake column represent the daily consumption level, categorized into 7 levels: 0: No intake 1: 10-20% intake 2: 30% intake 3: 40% intake 4: 50-60% intake 5: 70-80% intake 6: 90-100% intake.

Supplementary Table 4. Information on the amount of anti epileptic drug and food intake for each patient

Pt ID		Preoperation	Day 1	Day 2	Day 3	Day 4	Day 5	Day 6	Day 7	Day 8	Day 9	Day 10	Day 11	Day 12	Day 13
	LEV	3000	1000	→	→	→	→	→	→	→	→	→	→	→	→
	PER	8	→	→	→	→	→	→	→	→	→	→	→	→	→
	LCM	200	→	→	→	→	→	→	→	→	→	→	→	→	→
	Breakfast	Main dish	4	6	6	6	6	6	6	6	6	6	6	6	6
4		Side dish	6	6	6	6	6	6	6	6	6	6	6	6	6
	Lunch	Main dish	6	6	6	6	6	6	6	6	6	6	6	6	6
		Side dish	6	6	6	6	6	6	6	6	6	6	6	6	6
	Dinner	Main dish	6	6	6	6	6	6	6	6	6	6	6	6	6
		Side dish	6	6	6	6	6	6	6	6	6	6	6	6	6
	LEV	2000	1500	2000	→	→	→	→	→	→	→	→	→	→	→
	PER	2	→	→	→	→	→	→	→	→	→	→	→	→	→
	LCM	100	100	200	200	→	→	→	→	→	→	→	→	→	→
	Breakfast	Main dish	1	1	2	6	6	6	6	6	6	6	6	6	6
5		Side dish	1	2	2	6	6	6	6	6	6	6	6	6	6
	Lunch	Main dish	6	0	6	6	6	6	6	6	6	NaN	6	6	6
		Side dish	5	2	6	6	6	6	6	6	6	NaN	6	6	6
	Dinner	Main dish	4	6	6	6	6	6	6	6	6	6	6	6	6
		Side dish	4	4	6	6	6	6	6	6	6	6	6	6	6
	LEV	2500	→	1500	500	→	→	→	→	750	1000	1500	1750	2000	
	PER	8	→	→	→	→	→	2	→	4	4	→	6	8	
	LTG	400	→	→	→	→	→	200	100	200	300	→	400	400	
	Breakfast	Main dish	6	6	6	6	6	6	6	6	6	6	6	NaN	
6		Side dish	6	6	6	6	6	6	6	6	6	6	6	NaN	
	Lunch	Main dish	6	6	2	4	6	6	6	6	6	6	6	6	
		Side dish	6	6	2	4	6	6	6	6	6	6	6	6	
	Dinner	Main dish	6	6	6	6	6	6	6	6	6	6	6	6	
		Side dish	6	6	6	6	6	6	6	6	6	6	6	6	

LEV: levetiracetam**,** LTG: lamotrigine, LCM: lacosamide, CLB: clobazam, CBZ: carbamazepine, VPA: valproic acid, ZNS: zonisamide, PER: perampanel**.** The numbers indicated in the antiepileptic drug column represent the daily dosage in milligrams (mg). The numbers indicated in the meal intake column represent the daily consumption level, categorized into 7 levels: 0: No intake 1: 10-20% intake 2: 30% intake 3: 40% intake 4: 50-60% intake 5: 70-80% intake 6: 90-100% intake.

Supplementary Table 5. Information on the amount of anti epileptic drug and food intake for each patient

Pt ID		Preoperation	Day 1	Day 2	Day 3	Day 4	Day 5	Day 6	Day 7	Day 8	Day 9	Day 10	Day 11	Day 12	Day 13	Pt ID
7	LEV	1000	750	250	0	750	500	250	→	750	500	250	→	→	0	500
	LTG	125	75	25	→	50	25	→	→	50	→	25	→	50	0	25
	Breakfast	Main dish	6	6	6	4	6	6	NaN	6	6	6	6	6	6	6
		Side dish	6	6	6	4	6	6	NaN	6	6	6	6	6	6	6
	Lunch	Main dish	6	6	6	3	6	6	6	6	6	6	6	6	6	6
		Side dish	6	6	6	4	6	6	6	6	6	6	6	6	6	6
	Dinner	Main dish	6	6	6	4	6	6	6	6	6	6	6	6	6	6
		Side dish	5	6	6	4	6	6	6	6	6	6	6	6	6	6
8	LCM	400	200	200	100	0	100	100	0	200	400					
	VPA	1000	600	400	400	200	200	300	100	400	800					
	CLB	10	5	5	0	0	0	5	0	0	20					
	Breakfast	Main dish	1	6	6	6	6	6	6	1	6					
		Side dish	1	6	6	6	6	6	6	1	6					
	Lunch	Main dish	5	6	6	6	6	6	6	6	6					
		Side dish	5	1	6	6	6	6	6	6	6					
	Dinner	Main dish	6	6	6	6	6	6	6	6	6					
		Side dish	6	6	6	6	6	6	6	6	6					
9	LEV	3000	1500	0	→	→	→	→	→	1500	3000	→	→	→	→	
	CBZ	500	500	→	→	→	→	→	→	→	→	→	→	→	→	
	Breakfast	Main dish	1	1	2	6	6	6	6	6	6	6	2	4	1	
		Side dish	1	1	2	6	6	4	2	2	9	6	2	6	1	
	Lunch	Main dish	1	2	4	4	6	6	4	6	NaN	6	NaN	6	NaN	
		Side dish	2	1	2	3	6	6	2	6	NaN	6	NaN	6	NaN	
	Dinner	Main dish	1	1	6	4	6	4	4	6	6	6	4	NaN	1	
		Side dish	1	1	5	4	4	3	2	6	1	6	6	NaN	1	
10	LEV	2250	2250	1500	1000	→	1750	2250	2250	→	→	→	→			
	VPA	800	800	→	→	→	→	→	→	→	→	→	→			
	LCM	400	400	→	→	→	→	→	→	→	→	→	→			
	PER	4	4	→	→	→	→	→	→	→	→	→	→			
	Breakfast	Main dish	2	6	6	4	4	6	6	6	6	2	6			
		Side dish	4	4	6	6	6	6	6	6	6	6	6			
	Lunch	Main dish	4	6	6	6	6	6	6	6	6	6	6			
		Side dish	3	3	6	6	6	6	6	6	6	6	6			
	Dinner	Main dish	4	5	6	6	6	6	6	5	6	6	6			
		Side dish	4	5	6	4	6	6	6	5	6	6	6			

LEV: levetiracetam**,** LTG: lamotrigine, LCM: lacosamide, CLB: clobazam, CBZ: carbamazepine, VPA: valproic acid, ZNS: zonisamide, PER: perampanel**.** The numbers indicated in the antiepileptic drug column represent the daily dosage in milligrams (mg). The numbers indicated in the meal intake column represent the daily consumption level, categorized into 7 levels: 0: No intake 1: 10-20% intake 2: 30% intake 3: 40% intake 4: 50-60% intake 5: 70-80% intake 6: 90-100% intake.

Supplementary Table 6. Information on meal start and end times for each patient

		Breakfast start	Breakfast end	Lunch start	Lunch end	Dinner start	Dinner end
Pt-01	Day 1	-	-	-	-	-	-
	Day 2	8:20:35	8:32:41	12:43:05	13:17:54	17:56:04	18:26:07
	Day 3	7:27:44	8:03:21	12:15:32	13:15:00	17:58:15	18:41:55
	Day 4	7:39:39	8:08:17	12:28:49	13:00:27	18:01:35	18:43:58
	Day 5	7:47:11	8:39:25	12:20:04	13:11:19	17:48:44	18:20:59
	Day 6	7:41:29	8:16:07	12:18:33	12:48:33	18:00:35	18:47:39
	Day 7	7:43:54	8:21:47	12:20:52	12:58:34	17:59:57	18:38:06
	Day 8	7:36:59	7:51:45	12:56:17	13:24:37	17:52:26	NaN
	Day 9	7:48:11	8:15:50	12:20:24	12:41:51	17:55:42	18:19:55
	Day 10	7:44:59	8:20:07	12:16:30	12:42:54	17:48:19	18:56:43
	Day 11	7:52:17	8:18:50	-	-	-	-
Pt-02	Day 1	-	-	12:17:41	12:39:41	17:47:39	18:21:50
	Day 2	8:07:28	8:17:52	12:13:52	12:31:41	17:47:56	18:17:16
	Day 3	NaN	8:01:33	12:13:53	12:47:09	17:47:01	18:17:00
	Day 4	7:27:15	7:58:40	12:14:40	12:34:56	17:51:34	NaN
	Day 5	7:32:53	7:55:32	12:12:29	12:33:17	17:50:35	18:20:58
	Day 6	7:27:35	7:50:01	12:12:32	12:39:08	17:48:38	18:24:11
	Day 7	7:23:44	7:58:50	12:15:39	12:40:33	18:06:08	18:29:59
	Day 8	7:32:18	7:56:26	12:11:43	12:25:01	18:00:58	18:33:03
	Day 9	7:35:06	8:19:38	-	-	-	-
Pt-03	Day 1	-	-	12:15:32	12:45:47	17:50:31	18:15:29
	Day 2	7:24:45	7:56:7	NaN	NaN	17:50:40	18:14:34
	Day 3	7:31:53	7:51:47	12:12:32	12:39:34	NaN	NaN
	Day 4	7:28:10	7:50:52	12:14:44	12:41:07	17:53:05	18:23:11
	Day 5	7:26:19	7:42:24	12:18:12	12:39:18	17:46:24	18:16:55
	Day 6	7:26:09	7:43:19	12:15:11	12:34:58	17:52:48	18:17:36
	Day 7	7:31:57	7:49:15	12:16:05	12:40:06	17:45:46	18:12:13
	Day 8	7:25:29	7:40:08	12:20:00	12:40:58	17:45:26	18:06:36
	Day 9	7:25:32	7:41:55	NaN	NaN	NaN	NaN
	Day 10	NaN	NaN	12:18:38	12:38:57	17:50:24	18:21:39
	Day 11	7:25:39	7:44:24	-	-	-	-
Pt-04	Day 1	-	-	12:22:48	12:44:55	17:45:38	18:08:22
	Day 2	7:27:01	7:38:05	12:16:13	12:35:26	17:55:15	18:12:58
	Day 3	7:23:16	7:38:40	12:25:31	12:37:40	17:51:05	18:11:57
	Day 4	7:29:07	7:41:58	12:18:06	12:34:24	17:51:39	18:12:23
	Day 5	7:28:59	7:46:37	12:25:46	12:39:20	17:55:58	18:11:57
	Day 6	7:33:13	7:47:14	12:17:06	12:36:10	17:49:01	18:07:39
	Day 7	7:27:53	7:40:22	NaN	NaN	17:50:01	18:03:27
	Day 8	7:25:44	7:40:42	12:20:54	12:33:37	-	-

Supplementary Table 7. Information on meal start and end times for each patient. Video recording to annotate mealtime were not available in Pt-6.

		Breakfast start	Breakfast end	Lunch start	Lunch end	Dinner start	Dinner end
Pt-05	Day 1	-	-	12:49:34	13:15:02	NaN	NaN
	Day 2	NaN	NaN	NaN	NaN	NaN	NaN
	Day 3	NaN	NaN	12:20:00	12:42:51	NaN	NaN
	Day 4	NaN	NaN	12:24:41	12:53:55	NaN	NaN
	Day 5	NaN	7:55:40	12:21:24	12:47:04	NaN	NaN
	Day 6	NaN	NaN	12:23:18	12:44:37	NaN	NaN
	Day 7	NaN	7:56:18	12:23:28	12:43:52	NaN	NaN
	Day 8	NaN	NaN	12:29:48	12:47:04	NaN	NaN
	Day 9	NaN	NaN	12:14:06	12:28:32	18:04:18	18:42:29
	Day 10	7:49:16	8:06:06	12:23:40	12:38:23	NaN	NaN
	Day 11	NaN	NaN	14:00:22	14:18:03	-	-
Pt-07	Day 1	-	-	12:29:48	NaN	16:53:51	17:03:38
	Day 2	7:33:06	7:48:06	12:19:27	12:29:36	17:46:25	18:05:28
	Day 3	NaN	NaN	12:39:48	12:56:29	17:49:22	18:04:15
	Day 4	8:21:53	8:27:01	12:59:22	13:09:18	17:53:21	18:08:27
	Day 5	7:29:30	7:49:02	12:29:31	12:47:32	18:04:12	18:31:52
	Day 6	7:24:41	7:44:10	12:17:37	12:38:56	17:54:33	18:22:44
	Day 7	7:30:44	7:51:43	12:22:05	12:48:48	17:50:17	18:06:09
	Day 8	7:19:07	7:36:13	12:17:55	12:37:44	20:42:28	20:51:04
	Day 9	7:27:00	7:45:43	12:22:45	12:47:21	17:55:41	18:15:49
	Day 10	7:23:16	7:38:28	12:24:52	12:45:51	17:52:59	18:11:45
	Day 11	7:29:59	7:44:39	12:19:11	12:31:34	17:48:52	18:07:35
	Day 12	7:17:05	7:36:13	12:17:48	12:35:52	17:53:04	18:18:03
	Day 13	7:18:46	7:33:16	12:16:25	12:45:50	17:55:14	18:18:01
	Day 14	7:27:59	7:43:11	12:15:56	12:35:53	17:49:37	18:13:00
	Day 15	7:34:27	7:46:46	-	-	-	-
Pt-08	Day 1	-	-	-	-	17:53:06	18:02:09
	Day 2	7:25:04	7:34:24	12:22:06	12:34:46	18:01:52	18:13:12
	Day 3	7:27:15	7:36:35	12:25:07	12:36:34	17:51:15	17:59:56
	Day 4	7:23:49	7:31:23	12:21:23	12:33:37	17:58:28	18:16:29
	Day 5	7:30:55	7:51:49	12:25:38	12:37:52	NaN	NaN
	Day 6	NaN	NaN	12:26:45	12:39:59	17:57:12	18:07:10
	Day 7	7:28:45	7:40:00	12:24:03	12:34:44	18:20:39	18:30:26
	Day 8	9:16:22	9:25:29	12:16:05	12:26:23	18:40:40	18:56:27
	Day 9	7:42:05	7:48:20	12:24:13	12:33:18	-	-
Pt-09	Day 1	8:10:37	8:17:07	NaN	NaN	NaN	NaN
	Day 2	7:25:23	7:32:51	12:26:32	12:37:29	17:48:35	18:04:07
	Day 3	7:21:08	7:35:27	12:28:18	12:42:53	17:54:27	18:11:30
	Day 4	7:23:55	7:25:29	12:24:25	12:33:21	17:52:56	18:02:32

	Day 5	7:31:57	7:44:12	12:32:29	12:44:09	17:57:55	18:09:00
	Day 6	7:37:12	7:46:57	12:23:39	12:28:47	19:15:52	19:21:46
	Day 7	7:40:27	7:49:40	12:30:04	12:35:56	18:02:41	18:09:44
	Day 8	7:29:07	7:48:22	-	-	-	-
	Day 1	-	-	12:42:12	13:34:40	18:04:59	18:37:40
	Day 2	7:30:18	8:17:45	12:26:19	12:43:40	NaN	NaN
	Day 3	7:36:22	7:54:22	12:24:49	12:40:25	17:58:22	18:10:48
	Day 4	7:29:06	7:37:20	12:24:54	12:39:08	NaN	NaN
Pt-10	Day 5	7:28:54	7:48:28	12:23:36	12:37:28	17:56:18	18:19:38
	Day 6	7:24:51	7:37:36	12:20:12	12:29:56	18:44:09	18:56:00
	Day 7	7:30:27	7:48:00	12:24:27	12:40:47	18:32:48	18:43:46
	Day 8	7:25:59	7:38:03	12:35:25	12:48:46	19:54:01	20:06:51
	Day 9	7:29:54	7:42:13	12:28:00	12:37:34	-	-

Supplementary Table 8. Information on meal start and end times for each patient. Video recording to annotate mealtime were not available in Pt-6.

		Breakfast start	Breakfast end	Lunch start	Lunch end	Dinner start	Dinner end
	Day 1	-	-	12:49:34	13:15:02	NaN	NaN
	Day 2	NaN	NaN	NaN	NaN	NaN	NaN
	Day 3	NaN	NaN	12:20:00	12:42:51	NaN	NaN
	Day 4	NaN	NaN	12:24:41	12:53:55	NaN	NaN
	Day 5	NaN	7:55:40	12:21:24	12:47:04	NaN	NaN
Pt-05	Day 6	NaN	NaN	12:23:18	12:44:37	NaN	NaN
	Day 7	NaN	7:56:18	12:23:28	12:43:52	NaN	NaN
	Day 8	NaN	NaN	12:29:48	12:47:04	NaN	NaN
	Day 9	NaN	NaN	12:14:06	12:28:32	18:04:18	18:42:29
	Day 10	7:49:16	8:06:06	12:23:40	12:38:23	NaN	NaN
	Day 11	NaN	NaN	14:00:22	14:18:03	-	-
	Day 1	-	-	12:29:48	NaN	16:53:51	17:03:38
	Day 2	7:33:06	7:48:06	12:19:27	12:29:36	17:46:25	18:05:28
	Day 3	NaN	NaN	12:39:48	12:56:29	17:49:22	18:04:15
	Day 4	8:21:53	8:27:01	12:59:22	13:09:18	17:53:21	18:08:27
	Day 5	7:29:30	7:49:02	12:29:31	12:47:32	18:04:12	18:31:52
	Day 6	7:24:41	7:44:10	12:17:37	12:38:56	17:54:33	18:22:44
	Day 7	7:30:44	7:51:43	12:22:05	12:48:48	17:50:17	18:06:09
Pt-07	Day 8	7:19:07	7:36:13	12:17:55	12:37:44	20:42:28	20:51:04
	Day 9	7:27:00	7:45:43	12:22:45	12:47:21	17:55:41	18:15:49
	Day 10	7:23:16	7:38:28	12:24:52	12:45:51	17:52:59	18:11:45
	Day 11	7:29:59	7:44:39	12:19:11	12:31:34	17:48:52	18:07:35
	Day 12	7:17:05	7:36:13	12:17:48	12:35:52	17:53:04	18:18:03
	Day 13	7:18:46	7:33:16	12:16:25	12:45:50	17:55:14	18:18:01
	Day 14	7:27:59	7:43:11	12:15:56	12:35:53	17:49:37	18:13:00
	Day 15	7:34:27	7:46:46	-	-	-	-
	Day 1	-	-	-	-	17:53:06	18:02:09
	Day 2	7:25:04	7:34:24	12:22:06	12:34:46	18:01:52	18:13:12
	Day 3	7:27:15	7:36:35	12:25:07	12:36:34	17:51:15	17:59:56
	Day 4	7:23:49	7:31:23	12:21:23	12:33:37	17:58:28	18:16:29
Pt-08	Day 5	7:30:55	7:51:49	12:25:38	12:37:52	NaN	NaN
	Day 6	NaN	NaN	12:26:45	12:39:59	17:57:12	18:07:10
	Day 7	7:28:45	7:40:00	12:24:03	12:34:44	18:20:39	18:30:26
	Day 8	9:16:22	9:25:29	12:16:05	12:26:23	18:40:40	18:56:27
	Day 9	7:42:05	7:48:20	12:24:13	12:33:18	-	-
	Day 1	8:10:37	8:17:07	NaN	NaN	NaN	NaN
Pt-09	Day 2	7:25:23	7:32:51	12:26:32	12:37:29	17:48:35	18:04:07
	Day 3	7:21:08	7:35:27	12:28:18	12:42:53	17:54:27	18:11:30
	Day 4	7:23:55	7:25:29	12:24:25	12:33:21	17:52:56	18:02:32

	Day 5	7:31:57	7:44:12	12:32:29	12:44:09	17:57:55	18:09:00
	Day 6	7:37:12	7:46:57	12:23:39	12:28:47	19:15:52	19:21:46
	Day 7	7:40:27	7:49:40	12:30:04	12:35:56	18:02:41	18:09:44
	Day 8	7:29:07	7:48:22	-	-	-	-
	Day 1	-	-	12:42:12	13:34:40	18:04:59	18:37:40
	Day 2	7:30:18	8:17:45	12:26:19	12:43:40	NaN	NaN
	Day 3	7:36:22	7:54:22	12:24:49	12:40:25	17:58:22	18:10:48
	Day 4	7:29:06	7:37:20	12:24:54	12:39:08	NaN	NaN
Pt-10	Day 5	7:28:54	7:48:28	12:23:36	12:37:28	17:56:18	18:19:38
	Day 6	7:24:51	7:37:36	12:20:12	12:29:56	18:44:09	18:56:00
	Day 7	7:30:27	7:48:00	12:24:27	12:40:47	18:32:48	18:43:46
	Day 8	7:25:59	7:38:03	12:35:25	12:48:46	19:54:01	20:06:51
	Day 9	7:29:54	7:42:13	12:28:00	12:37:34	-	-
