# Supplementary material for: Hippocampal sharp-wave ripples correlate with periods of naturally occurring self-generated thoughts in humans
Source: Nat Commun. 2024 May 22;15:4078. doi: 10.1038/s41467-024-48367-1 (PMC11111804; doi:10.1038/s41467-024-48367-1)
Supplement: Supplementary file 3 — Reporting Summary [file 41467_2024_48367_MOESM3_ESM.pdf]

nature portfolio

Corresponding author(s): Takufumi Yanagisawa

Last updated by author(s): Mar 18, 2024

Reporting Summary

Nature Portfolio wishes to improve the reproducibility of the work that we publish. This form provides structure for consistency and transparency in reporting. For further information on Nature Portfolio policies, see our Editorial Policies and the Editorial Policy Checklist.

Statistics

For all statistical analyses, confirm that the following items are present in the figure legend, table legend, main text, or Methods section.

n/a	Confirmed	
☐	☒	The exact sample size (*n*) for each experimental group/condition, given as a discrete number and unit of measurement
☐	☒	A statement on whether measurements were taken from distinct samples or whether the same sample was measured repeatedly
☐	☒	The statistical test(s) used AND whether they are one- or two-sided<br>*Only common tests should be described solely by name; describe more complex techniques in the Methods section.*
☐	☒	A description of all covariates tested
☐	☒	A description of any assumptions or corrections, such as tests of normality and adjustment for multiple comparisons
☐	☒	A full description of the statistical parameters including central tendency (e.g. means) or other basic estimates (e.g. regression coefficient) AND variation (e.g. standard deviation) or associated estimates of uncertainty (e.g. confidence intervals)
☐	☒	For null hypothesis testing, the test statistic (e.g. *F*, *t*, *r*) with confidence intervals, effect sizes, degrees of freedom and *P* value noted<br>*Give P values as exact values whenever suitable.*
☒	☐	For Bayesian analysis, information on the choice of priors and Markov chain Monte Carlo settings
☒	☐	For hierarchical and complex designs, identification of the appropriate level for tests and full reporting of outcomes
☒	☐	Estimates of effect sizes (e.g. Cohen's *d*, Pearson's *r*), indicating how they were calculated

Our web collection on statistics for biologists contains articles on many of the points above.

Software and code

Policy information about availability of computer code

Data collection	We recorded intracranial EEG data from subdural electrodes and depth electrodes sampled at 10 kHz with an EEG-1200 (Nihon Kohden, Tokyo, Japan). We simultaneously recorded subjects' physiological states, such as electrodermal activity (EDA), three-dimensional acceleration data, blood volume pulse (BVP), and the interbeat interval (IBI), with the Empatica E4 wristband (Empatica, Milan, Italy). Subjects' mood and thought contents were measured by a tablet-based self-report questionnaire composed of 17 questions.<br>The code written to detect SWRs is available at https://doi.org/10.6084/m9.figshare.22815746.v1.
Data analysis	Collected data was analyzed using a program originally created in Matlab 2017b.

For manuscripts utilizing custom algorithms or software that are central to the research but not yet described in published literature, software must be made available to editors and reviewers. We strongly encourage code deposition in a community repository (e.g. GitHub). See the Nature Portfolio guidelines for submitting code & software for further information.

Data

Policy information about availability of data

All manuscripts must include a data availability statement. This statement should provide the following information, where applicable:

- Accession codes, unique identifiers, or web links for publicly available datasets
- A description of any restrictions on data availability
- For clinical datasets or third party data, please ensure that the statement adheres to our policy

The raw LFP data are available under restricted access as they contain information that could compromise the patients' privacy, and consent for their publication was not obtained. However, access can be obtained by reasonable request to the corresponding author, T.Y.
The SWR event rates, cortical delta power, and thought sampling data generated and analyzed in the present study have been deposited in Figshare at https://doi.org/10.6084/m9.figshare.22633369.v1.
The remainder of the data shown in the figures are provided in the Source Data file.
Source data are provided with this paper.

Research involving human participants, their data, or biological material

Policy information about studies with human participants or human data. See also policy information about sex, gender (identity/presentation), and sexual orientation and race, ethnicity and racism.

Reporting on sex and gender	Sex was not considered in the study design and sex of participants was determined based on self-report. Our data includes 6 males and 4 females.
Reporting on race, ethnicity, or other socially relevant groupings	The socially constructed or socially relevant categorization variables were not used in our manuscript.
Population characteristics	This study consist of eleven patients (self-reported sex: 6 males, 4 females; age: 32.8 ± 13.8 years) who were diagnosed as refractory epilepsy.
Recruitment	23 patients with drug-resistant epilepsy underwent intracranial electrode implantation for presurgical evaluation of seizure onset zones and memory function between January 2020 and May 2022. Of these, 15 patients (16 electrodes) had depth electrodes implanted in the hippocampus or parahippocampal gyrus. To minimize the influence of epileptic activity, recordings were excluded from the analysis if the hippocampus was pathologically diagnosed with hippocampal sclerosis. Ultimately, ten patients with eleven electrodes (self-reported sex: 6 males, 4 females; age: 33.6 ± 14.2 years; mean ± standard deviation [SD]; see Supplementary Table 1) who consented to participate in this study (i.e., to complete the questionnaire and wear a wearable device) were included. Electrode placement was determined solely by clinical necessity. In all patients, the clinical team determined the placement of the electrodes to best localize epileptogenic regions. To minimize the selection bias, the EEG data analyst did not participate in the determination of electrode placement. It is possible that selection bias could be introduced by not including data from patients who were uncooperative in responding to the questionnaire or wearing the wearable device.
Ethics oversight	The research protocol was approved by the Institutional Review Board (approval no. 14353, UMIN000017900), and informed consent was obtained from the participants.

Note that full information on the approval of the study protocol must also be provided in the manuscript.

Field-specific reporting

Please select the one below that is the best fit for your research. If you are not sure, read the appropriate sections before making your selection.

☒ Life sciences ☐ Behavioural & social sciences ☐ Ecological, evolutionary & environmental sciences

For a reference copy of the document with all sections, see nature.com/documents/nr-reporting-summary-flat.pdf

Life sciences study design

All studies must disclose on these points even when the disclosure is negative.

Sample size	To conduct the regression analysis, we predetermined the total number of answers on questionnaires as more than 170 samples, which is equivalent to 10 times the number of questionnaire items, totalling 17. The ratio of sample size to the number of parameters in the regression analysis was determined based on previous studies. The amount of data collected per patient depended on their clinical treatment schedule and the amount of time each participant was willing to volunteer for the study.
Data exclusions	To minimize the influence of epileptic activity, the recording from the hippocampus that were pathologically diagnosed with hippocampal sclerosis were not included in the analysis. Finally, the eleven patients with eleven electrodes who consented to participate in this study (i.e., completed the questionnaire and wear a wearable device) were included.

Replication	To ensure reproducibility, a nested cross-validation was carried out on 10 subject data, and learning and testing was carried out on different subject data to confirm that the results could be reproduced even when learning was based on different subjects.
Randomization	The study was observational and not an intervention study, therefore randomization was not performed.
Blinding	This study was a single-arm observational study, therefore the investigators are not blinded to group allocations. But, the patients and investigators were blinded to the SWR event when the patients reported on the questionnaire.

Reporting for specific materials, systems and methods

We require information from authors about some types of materials, experimental systems and methods used in many studies. Here, indicate whether each material, system or method listed is relevant to your study. If you are not sure if a list item applies to your research, read the appropriate section before selecting a response.

Materials & experimental systems

n/a	Involved in the study
☒	☐ Antibodies
☒	☐ Eukaryotic cell lines
☒	☐ Palaeontology and archaeology
☒	☐ Animals and other organisms
☐	☒ Clinical data
☒	☐ Dual use research of concern
☒	☐ Plants

Methods

n/a	Involved in the study
☒	☐ ChIP-seq
☒	☐ Flow cytometry
☒	☐ MRI-based neuroimaging

Clinical data

Policy information about clinical studies

All manuscripts should comply with the ICMJE guidelines for publication of clinical research and a completed CONSORT checklist must be included with all submissions.

Clinical trial registration	UMIN000017900
Study protocol	https://center6.umin.ac.jp/cgi-open-bin/ctr_e/ctr_view.cgi?recptno=R000019985
Data collection	In our hospital, 23 patients with drug-resistant epilepsy underwent intracranial electrode implantation for presurgical evaluation of seizure onset zones and memory function between January 2020 and May 2022. Of these, 15 patients (16 electrodes) had depth electrodes implanted in the hippocampus or parahippocampal gyrus. To minimize the influence of epileptic activity, recordings were excluded from the analysis if the hippocampus was pathologically diagnosed with hippocampal sclerosis. Ultimately, ten patients with eleven electrodes (self-reported sex: 6 males, 4 females; age: 33.6 ± 14.2 years; mean ± standard deviation [SD]; see Supplementary Table 1) who consented to participate in this study (i.e., to complete the questionnaire and wear a wearable device) were included. Electrode placement was determined solely by clinical necessity. In all patients, the clinical team determined the placement of the electrodes to best localize epileptogenic regions. The EEG data analyst did not participate in the determination of electrode placement. Data were collected at the Department of Neurosurgery at Osaka University Hospital.
Outcomes	We assessed intracranial EEG.

Plants

Seed stocks	n.a.
Novel plant genotypes	n.a.
Authentication	n.a.
